# Toward the Treatment of Glioblastoma Tumors Using Photoactivated Chemotherapy: In Vitro Evaluation of Efficacy and Safety

**DOI:** 10.1021/acsptsci.4c00600

**Published:** 2025-01-30

**Authors:** Sina Katharina Goetzfried, Matthijs L. A. Hakkennes, Anja Busemann, Sylvestre Bonnet

**Affiliations:** Leiden Institute of Chemistry, Leiden University, Einsteinweg 55, 2333CC Leiden, The Netherlands

**Keywords:** photopharmacology, glioblastoma, cancer, neurotoxicity, photoactivated chemotherapy, ruthenium, metallodrugs

## Abstract

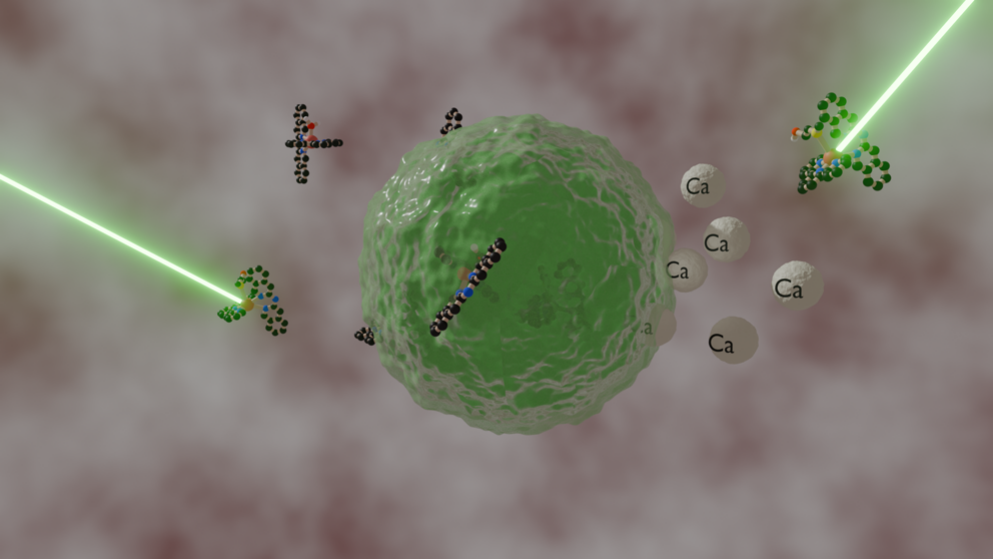

Glioblastoma multiforme (GBM) is highly aggressive, necessitating new therapies. Photoactivated chemotherapy (PACT) offers a promising approach by activating prodrugs with visible light at the tumor site. This study evaluated the anticancer activity of ruthenium-based PACT compounds in U-87MG glioblastoma cells and their safety in SH-SY5Y neuron-like cells. The compound [**3**](PF_6_)_2_ showed promising light-activated anticancer effects in U-87MG cells, while [**1**](PF_6_)_2_ was inactive, and [**2**](PF_6_)_2_ was nonactivated. Interestingly, in SH-SY5Y cells, light-activated [**3**](PF_6_)_2_ increased cell proliferation, similar to donepezil, without causing cell death. Increased Ca^2+^ uptake was observed, possibly via interaction with the AMPA receptor, as suggested by docking studies. These findings suggest ruthenium-based PACT compounds may serve as potential treatments for GBM, effectively attacking cancer cells while preserving healthy neuronal cells.

Albeit glioblastoma multiforme (GBM) is one of the most common malignant primary brain tumors, its treatment continues to challenge the medical community today.^1,2^ Each year, more than 250,000 new cases occur worldwide, while 200,000 patients die from this cancer, with an annual growth rate of 1.5%.^3^ According to recent studies, the incidence has increased from 0.73 to 4.49 per 100,000 people in the last 10 years worldwide.^4^ Despite having a lower incidence compared to other cancers such as lung, breast, or prostate cancer, GBM has a survival rate of 14–16 months and a five-year overall survival rate of less than 10%.^5^ Novel therapeutic modalities are urgently needed.

Nowadays, treatment options are limited by the location, phenotype,^6,7^ and aggressiveness of the tumor.^8^Figure 1B gives a schematic overview of the current problems in GBM treatment. Craniotomy, which consists of the surgical removal of the tumor, is still the first-line treatment, followed by radiation or chemotherapy.^9^ Only a few chemotherapeutics received approval for the treatment of GBM, namely,^10^ lomustine,^11^ intravenous carmustine, carmustine wafer implants,^12^ temozolomide,^13^ and the antibody bevacizumab.^14^ The most challenging aspect of chemotherapy in GBM treatment is that many drugs cannot cross the blood–brain barrier (BBB), which prevents reaching tumors inside the brain and causes neurological side effects. This was notably observed for bortezomib, which failed in early clinical trials^15^ or showed no significant improvements compared to current treatment methods.^15,16^ Another issue is the heterogeneity of brain tumors, which leads to very different responses of GBM cells to most known drugs, including resistances.^17−19^ Despite past drug development efforts, recurrence postsurgery is almost systematic, and patients suffer during treatment from severe side effects, including nausea, vomiting, seizures, and troubles with speech or memory.^20−24^

**Figure 1:**
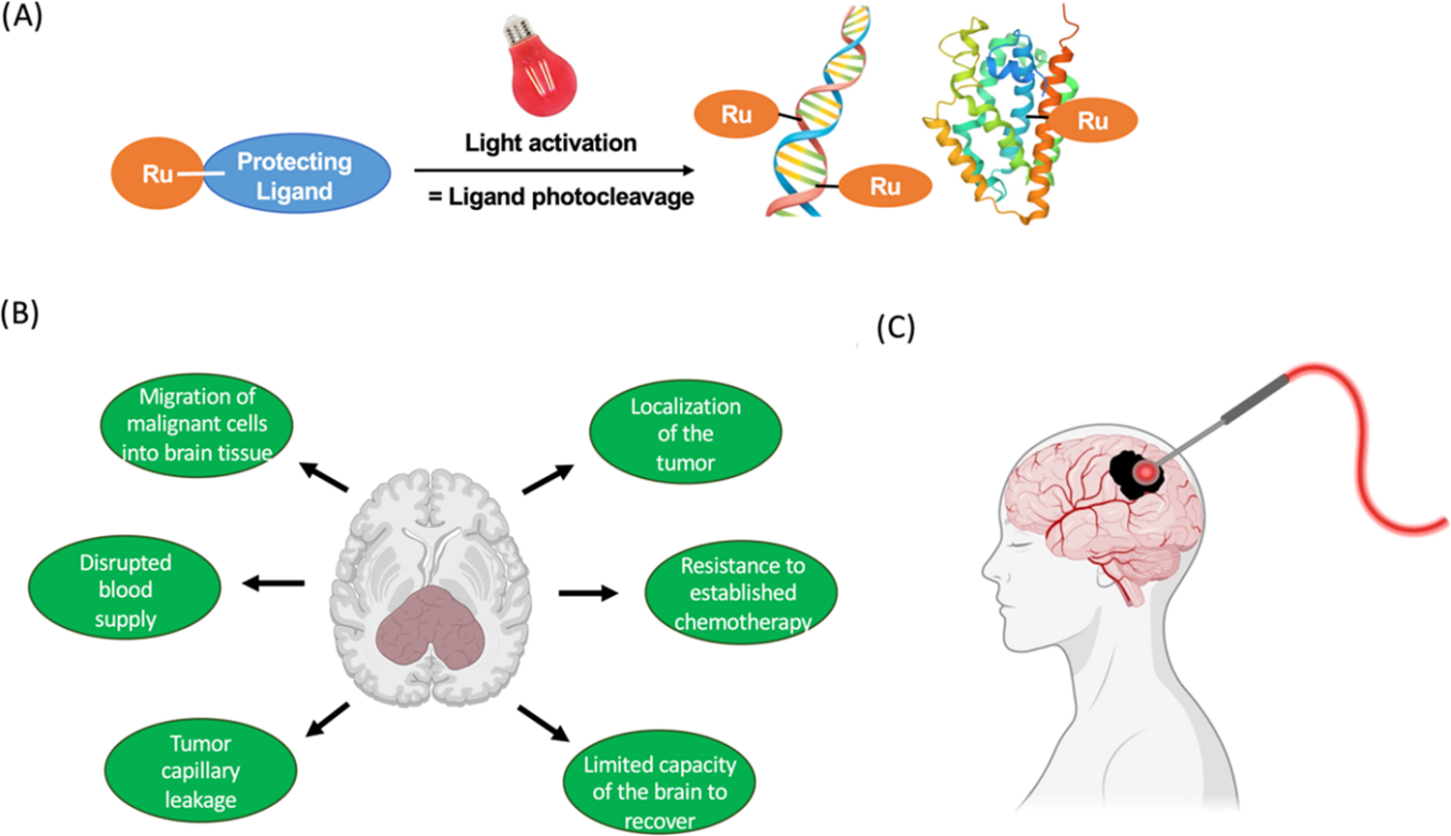
(A) Principle of ruthenium-based photoactivated chemotherapy (PACT). (B) Schematic overview of the challenges in GBM treatment. (C) Schematic drawing of the light balloon used in GBM treatment with 5-aminolevulinic acid. More detailed clinical illustrations can be obtained in ref (25). The figure was partly created by biorender.com.

5-Aminolevulinic acid (5-ALA) is a new agent that recently brought some light at the end of the tunnel.^26^ It is a small molecule that does cross the BBB in high-grade GBM tumors. It then accumulates in malignant tissue, where it is converted into protoporphyrin IX (PPIX), a naturally occurring porphyrin that shows strong luminescence properties. This molecule can be used for fluorescence-guided surgery in GBM resection (Figure 1C).^25,27−29^ 5-ALA also opened the door to light therapy for brain tumor treatment.^30−32^ PPIX indeed has excellent properties as a photosensitizer for photodynamic therapy (PDT). PDT drugs are photocatalysts that convert, under the action of visible light, dioxygen to singlet oxygen (^1^O_2_) and/or other reactive oxygen species (ROS).^33,34^ Shining light in the brain post 5-ALA administration can be performed following two main approaches depending on whether the tumor can be surgically removed or not. For patients who can be operated, a balloon is inserted postsurgery into the tumor cavity. This balloon is full of a light-scattering material that homogeneously distributes light onto the surface of the tumor cavity. The idea here is to “clean” the border of the tumor cavity and kill all cancer cells that may have remained from incomplete tumor ablation. The second light irradiation method is for patients whose light irradiation cannot be operated. In such a case, interstitial phototherapy is preferred:^35^ at least two small holes are made in the skull to insert two optical fibers in the core of the tumor in the brain. Both fibers are terminated by a light-diffusing end that scatters light radially. One is used for shining light into the tumor tissue, and the other one is used as a sensor to detect the light dose deposited into the brain tissue. Surgeons may swap both fibers to ensure proper light deposition into the tumor.^25,35^ Although 5-ALA PDT has shown excellent results in clinical phase I and is now in phase II, protoporphyrin IX is only overexpressed in grade III and IV tumors and is a PDT type II sensitizer, capable of generating ^1^O_2_ only in oxygen-rich regions of tumors. 5-ALA PDT of glioblastoma has shown promising clinical results in nonresectable and postresection GBM treatment.^36,37^ On the other hand, PDT agents are known to cause necrosis in the surrounding area and are limited by the need for high oxygen concentrations. In addition, overexpression of PPIX in patients treated with 5-ALA only takes place in grade III and IV glioblastoma. There is hence still a need for new phototherapeutic treatment modalities that rely less on the presence of oxygen to be activated and that may cross the blood–brain barrier in grade I and II glioblastoma.^38−39,40^

Photoactivated chemotherapy (PACT), a recently developed phototherapeutic modality, may overcome these problems. PACT drugs are “photocaged” compounds consisting of two different components fused into a single prodrug that is biologically inactive. After exposure to light, the two molecules split, thereby recovering their anticancer properties. This method raises interest, as it should not only lower side effects for patients like in PDT but also protect the underlying healthy tissues from necrosis, which is often observed in clinical PDT. Ru^2+^- or Pt^4+^-based PACT compounds are the most developed PACT agents because the photochemistry of these two metal centers allows a coordination bond to be selectively broken, either by photosubstitution for Ru^2+^ or by photoreduction for Pt^4+^.^13^ Typically, photochemical bond cleavage generates free organic inhibitors; however, the Ru-containing photoproduct can also generate phototoxicity by interacting with various biological targets (Figure 1A).^36,37^ The photosubstitution mechanism involves thermal promotion from the excited metal-to-ligand charge transfer excited state (^3^MLCT) generated photochemically to a dissociative, low-lying triple metal center state (^3^MC), leading to elongation of a bond and further dissociation of the ligand, without changing the oxidation state of the metal center.^41,42^

Over the last few decades, different ruthenium-based PACT compounds have been proposed for the treatment of different kinds of tumors.^43−51^ Despite their great potential, the use of ruthenium-based compounds to treat GBMs has, however, rarely been reported.^52−54^ Although in PACT, the Ru polypyridine compounds used as photocages have often been reported as biologically inactive,^50,55^ it should also not be ignored that drugs may be toxic to brain tissues adjacent to the targeted tumor and therefore may cause neuro- or excitotoxicity in an undesirable manner. Heavy metal elements often have a bad reputation for their effects on health, but in fact, little data are available on the pharmacology and toxicity of ruthenium polypyridyl complexes on the brain.

Here, we aimed to fill this knowledge gap by investigating in vitro if ruthenium-based PACT compounds may cause potential neuro- or excitotoxic effects that might limit their use for glioblastoma treatment. For this purpose, a series of known ruthenium-based PACT complexes with the general formula [Ru(tpy)(N^∧^N)(Hmte)](PF_6_)_2_ ((tpy: 2,2′;6′,2″-terpyridine; Hmte = 2-methylthioethanol; and N^∧^N = bpy = 2,2′-bipyridine in [**1**](PF_6_)_2_), N^∧^N = *i*-biq = bisisoquinoline in [**2**](PF_6_)_2_, N^∧^N = *i*-diqa = di(isoquinolin-3-yl)amine in [**3**](PF_6_)_2_, see Scheme 1)^56^ were reinvestigated in vitro in cell lines relevant for glioblastoma. We focused our study on the following: On the one hand, the impact of these complexes on cancer cell proliferation using not only the classical GBM cell line U-87MG but also the neuronal-like, “healthy” cell line SH-SY5Y. On the other hand, we aimed to study the effects of these compounds, either in the dark or upon green light irradiation, on the intracellular Ca^2+^ level in the healthy SH-SY5Y cells, which may point to neuro- or excitotoxic effects. To ensure that the biological response is triggered exclusively by the ruthenium-based activated product, we used here complexes that photosubstitute a biological inactive ligand (Hmte).^56^ Overall, the biological experiments proposed in this study represent a first methodological step toward a more complete in vivo safety evaluation of the potential side effects of GBM treatment using ruthenium-based PACT.

**Scheme 1:**
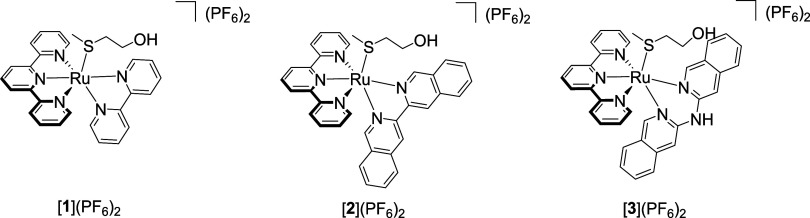
Chemical Structure of Complexes [**1**](PF_6_)_2_-[**3**](PF_6_)_2_ Used in This Study

## Results

### Stability in the Dark in the Presence of Nucleophiles

The synthesis and characterization of all three complexes have been described earlier and can be found in refs (56) and (57). In water or OptiMEM cell medium, the complexes were also previously found to be stable in the dark for at least 24 h at 37 °C. Upon blue or green light irradiation (λ = 452 or 517 nm), they are activated by photosubstitution of the thioether ligand to afford the aqua ruthenium complex and the free thioether ligand.^56^ In addition to previous stability investigations, we tested the stability in the dark of complexes [**1**](PF_6_)_2_–[**3**](PF_6_)_2_ in water in the presence of 500 μM l-glutathione (GSH), which is one of the main metal-deactivating thiols in living cells. We first recorded the UV–vis spectra of the respective complex in water at 37 °C (concentration 0.05 ([**1**](PF_6_)_2_) or 0.08 ([**2**](PF_6_)_2_ and [**3**](PF_6_)_2_) mM) and then added GSH at *t* = 0. The UV–vis spectra were then recorded after 2 and 24 h separately. As depicted in (Figure S1, Supporting Information), no change suggesting ligand exchange on ruthenium was observed for [**1**](PF_6_)_2_. For the more sterically hindered compounds [**2**](PF_6_)_2_ and [**3**](PF_6_)_2_, no isosbestic points suggesting ligand exchange were observed either, but we observed an increase of the absorbance spectrum versus time at short wavelengths. Additionally, we could not observe any color change, which is typical when one of the ligands is exchanged against another one. From these data, it seems unlikely that the monodentate thioester ligand is thermally dissociated from the metal center upon attack from glutathione.^58^

### Cytotoxicity and Cellular Uptake in the GBM Cell Line

With the aim to investigate the impact of the ruthenium PACT compound on GBM cells, compounds [**1**](PF_6_)_2_–[**3**](PF_6_)_2_ (Scheme 1) were incubated in U-87MG glioblastoma cells for 24 h at various concentrations, irradiated or not with green light (520 nm, 30 min, 25.2 J/cm^2^) without exchanging the medium, and further incubated for 48 h, before measuring relative cell proliferation using a sulforhodamine B (SRB) end-point assay.^59,60^ [**1**](PF_6_)_2_ did not show any cytotoxic effect (Table 1 and Figure S2), which fits with earlier reports about the nontoxic character of [Ru(tpy)(bpy)(OH_2_)]^2+^ ([**4**]^2+^) in A375 skin melanoma or A549 lung cancer cell lines.^50,55,56^ However, increasing the aromatic surface of the complex and therefore the lipophilicity of the prodrug resulted in antiproliferative activity after light irradiation for [**2**](PF_6_)_2_ and [**3**](PF_6_)_2_ (Table 1 and Figure S2). For [**2**](PF_6_)_2_, no significant difference was observed between dark and green light-activated conditions (EC_50,D_ = 19 μM, EC_50,GL_ = 23 μM, PI = 0.8). The most efficient PACT complex was *i*-diqa-bearing complex [**3**](PF_6_)_2_, which showed a photo index (PI) of 3.5 (EC_50,D_ = 37 μM, EC_50,GL_ = 11 μM). It is noteworthy that our compounds were more efficient compared to the FDA-approved compound temozolomide, which is currently the first-line treatment for GBM.

**Table 1:** Cytoxicity (EC_50_ in μM) of [**1**](PF_6_)_2_–[**3**](PF_6_)_2_ in Glioblastoma U-87MG Cells after Being Kept in the Dark or after Green Light Irradiation (520 nm, 30 min, 25.2 J/cm^2^)^a^

compound	EC_50,D_ [μM]	CI_95_ [μM]	EC_50,GL_ [μM]	CI_95_ [μM]	PI
[**1**](PF_6_)_2_	>100		61.66		>1.6
[**2**](PF_6_)_2_	19.4	+32.8	23.3	+11.5	0.8
		–14.4		–8.5	
[**3**](PF_6_)_2_	37.0	+8.2	11.1	+4.4	3.4
		–7.1		–3.4	
donezepil HCl	>100		>100		
temozolimde^b^	>100		>100		

a Cytotoxic experiments were performed in normoxic conditions (21% O_2_) in biological and technical triplicates; errors indicate 95% confidence intervals (CIs) in μM.

b Determined by MTT (3 mg/mL).

**Figure 2:**
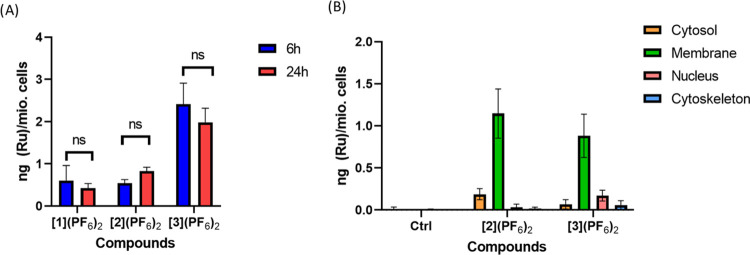
(A) Total Ru cellular uptake in U-87MG cells after 6 (blue bars) and 24 h (red bars) incubation time in the dark. (B) Ru content of different cell fractions after 24 h of incubation time. Ru concentrations: 10 μM.

The cellular accumulation of Ru^2+^ can provide additional information about how differences in anticancer efficacy may relate to the amount of the Ru prodrug in the cell before light activation. For instance, low uptake often leads to limited cytotoxicity due to limited access to the drug target. Therefore, we incubated [**1**](PF_6_)_2_–[**3**](PF_6_)_2_ (10 μM) in U-87MG cells during 6 or 24 h and then quantified the intracellular Ru content using inductively coupled plasma-mass spectrometry (ICP-MS). Figure 2A displays the metal content in ng Ru per million cells. After 6 h, 4 times lower amounts of [**1**](PF_6_)_2_ (0.6 ng Ru/mio. cells) and [**2**](PF_6_)_2_ (0.5 ng of (Ru)/mio. cells) were taken up compared with that measured for [**3**](PF_6_)_2_ (2.1 ng (Ru)/mio. cells, Table S1). These numbers did not change significantly after 24 h of incubation, and a slightly lower amount of [Ru] was found for [**1**](PF_6_)_2_ (0.42 ng (Ru)/mio. cells) and [**3**](PF_6_)_2_ (1.98 ng (Ru)/mio. cells). In terms of efficacy, the overall uptake was only 0.5% for [**1**](PF_6_)_2_ and 0.8 or 1.5% for [**2**](PF_6_)_2_, while almost 11.4 or 11.5% of [**3**](PF_6_)_2_ was taken up in U-87MG cells under such conditions. Similar results have been observed in A549 lung cancer cells previously.^56^ It is surprising to find such different uptake values for [**2**](PF_6_)_2_ and [**3**](PF_6_)_2_, as both compounds have similar LogP values (3.08 and 2.92, respectively).^56^ For unknown reasons, it appears that the amine bridge in the *i*-diqa ligand facilitates the uptake of ruthenium compound [**3**](PF_6_)_2_.

To further investigate in which compartment in the cells the ruthenium complexes localized before light activation, we used a FractionPREP Cell Fractionating Kit to separate the different compartments (cytosol, membrane, nucleus, and cytoskeleton) of the cells and analyzed the Ru content of all fractions by ICP-MS for U-87MG cells treated for 24 h with 10 μM [**2**](PF_6_)_2_ or [**3**](PF_6_)_2_ (Figure 2B and Table S2). [**1**](PF_6_)_2_ was excluded from this experiment, as it had poor cytotoxicity and showed a very low uptake in previous experiments. As depicted in Figure 2B, both [**2**](PF_6_)_2_ and [**3**](PF_6_)_2_ were mostly found in the membrane fraction (0.83 or 0.88 ng Ru/mio. cells). A small amount of [**2**](PF_6_)_2_ was found in the cytosol (0.18 ng Ru/mio. cells), while almost the same amount (0.17 ng Ru/mio. cells) was recorded for [**3**](PF_6_)_2_ in the nucleus. Almost no Ru was detected in the cytoskeleton of both complexes. As noted, those data only provide information about where the prodrug was localized before light activation, but it does not say anything about where the activated photoproducts go.

Within this series of structurally related compounds, several trends can be highlighted. First, [**1**](PF_6_)_2_ was poorly phototoxic due to its very low cellular uptake. Surprisingly, the exchange of the bpy ligand by *i*-biq increased cytotoxicity at least by a 3-fold factor, although uptake did not increase significantly; still, no difference between dark and light EC_50_ values was seen for this complex in this GBM cell line. The best light-activated figure-of-merit in this series was observed for [**3**](PF_6_)_2_. This complex became 3.5 times more toxic upon green light irradiation compared with dark conditions; compared with [**2**](PF_6_)_2_, it was twice less cytotoxic in the dark in spite of the 4 times higher uptake and twice more cytotoxic after light irradiation. In addition, the uptake and fractionation results are compatible with a membrane association of both active complexes and a poor cellular uptake before activation. As noted, it is impossible to image and colocalize ruthenium-based PACT complexes in a cell by confocal microscopy, as these compounds are nonemissive.

### In Vitro Approach to Neurotoxicity

With these results in hand, we wondered how to investigate in vitro the potential neurotoxicity of these compounds and hence their damaging properties toward adjacent, healthy neurons. Testing the neurodamaging effects of chemical compounds is a difficult venture, as it comes in hand with unique difficulties.^61−64^ Several in vivo characteristics, e.g., the low neurogenesis in adults, a great level of cellular, structural, and chemical heterogeneity, distinct metabolic requirements, and a large number of neuronal messengers, render the nervous system particularly vulnerable to damages induced by chemicals. When used for treating brain tumors, new chemotherapy compounds, even photoactivated ones, may potentially impair sensory and motor functions or interfere with the memory process, for example, which is difficult to test in vitro. However, some tests have been proposed, including the inhibition of different enzymes, such as acetylcholine esterase (AChE), γ-aminobutyric acid (GABA) receptors, *N*-methyl-d-aspartate (NMDA) receptors, or serotonin receptors. Last but not least, the neuroblastoma cell line SH-SY5Y has been proposed as a suitable cell line to evaluate the neuro- and excitotoxic effects of chemical compounds, usually not in cancer research but for developing drugs against neurodegenerative diseases.^65−69^

**Figure 3:**
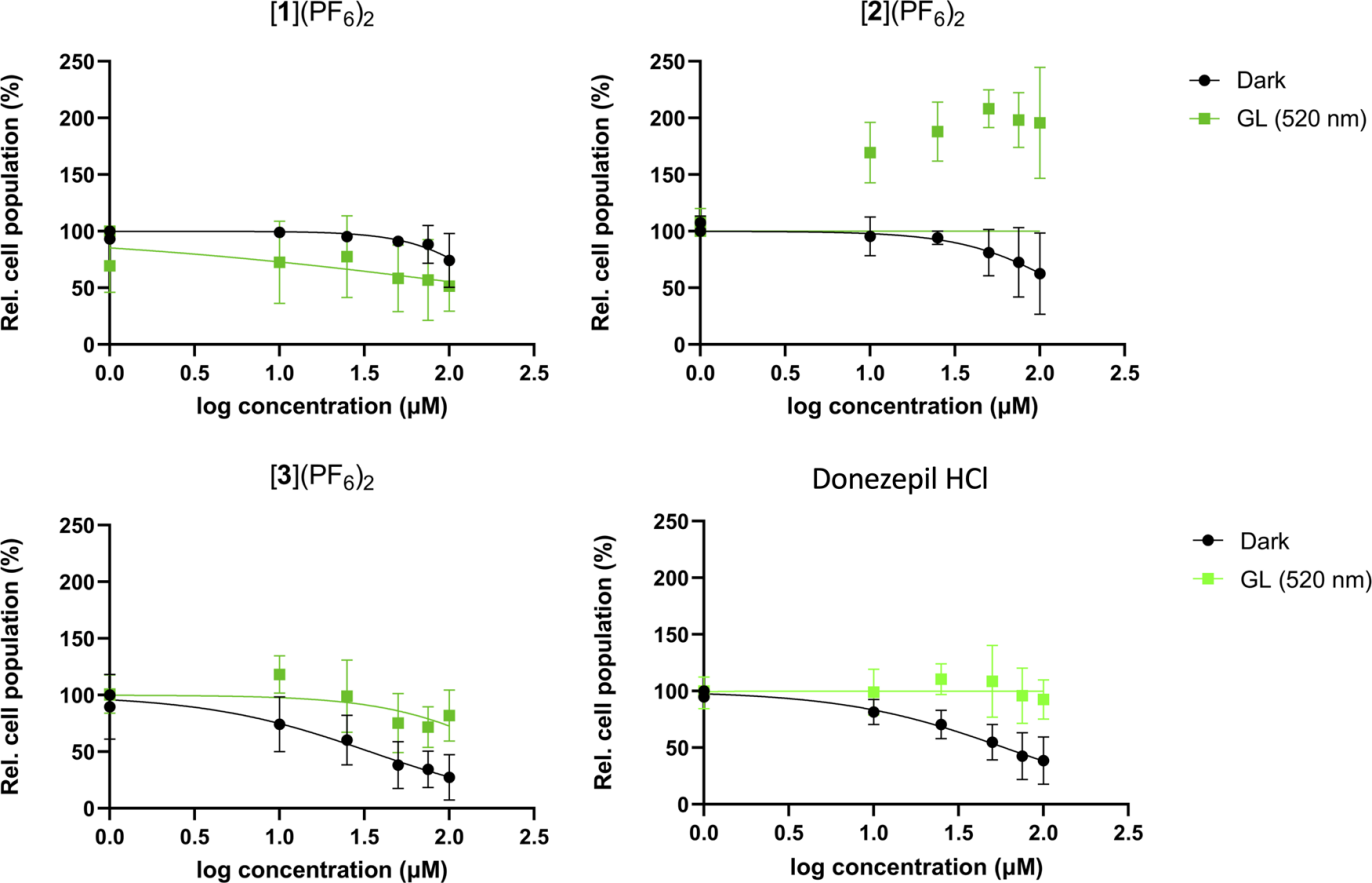
Dose–response curve of [**1**](PF_6_)_2_–[**3**](PF_6_)_2_ complexes and donepezil HCl in SH-SY5Y cells in the dark and upon green light activation (30 min, 520 nm, 25.2 J/cm^2^). Cells were irradiated with green light 24 h after compound administration.

Using the same protocol as for the U-87MG cancer cell line, we first investigated the cytotoxicity of [**1**](PF_6_)_2_–[**3**](PF_6_)_2_ in the sympathetic ganglion-neuron-like SH-SY5Y cell line. The HCl salt of the AChE inhibitor donepezil, donepezil HCl, was included in the study as a typical, clinically approved drug used for treating brain diseases such as Alzheimer’s disease.^70^ The cells were hence were incubated with increasing concentrations of each compounds 24 h in the dark, irradiated with light without changing the medium, and further incubated 48 h before the end-point SRB assay (Figure 3 and Table 2).^59,60^ In the dark, [**1**](PF_6_)_2_ and [**2**](PF_6_)_2_ required high concentrations (>40 μM) to block cell growth. Only [**3**](PF_6_)_2_ possessed EC_50_ values below 50 μM. Unexpectedly, when the ruthenium compounds were exposed to green light (520 nm, 30 min, 25.2 J/cm^2^), a reverse effect was observed compared with U-87MG cells: more protein was observed in the SRB assay than in the dark, suggesting an increased number of living cells at *t* = 96 h time point, up to 200% for [**2**](PF_6_)_2_ (Figure 3B). For [**3**](PF_6_)_2_, the end-point relative cell population remained close to 100%, suggesting that the limited toxicity observed in the dark had been completely canceled (Figure 3C). It is also worth noticing that green light alone had an impact on the growth of SH-SY5Y cells, as cells irradiated with light in the absence of the Ru compound showed reduced cell growth by ∼50% compared with cells kept in the dark. The ruthenium prodrugs hence seem to protect the cells against this effect. Our results in the absence of the compound confirmed previous studies on SH-SY5Y cells that had shown that green light irradiation influenced dendrite elongation and reduced cell growth.^71^ These observations were, surprisingly, similar to that of donepezil HCl (Figure 3D), which slightly reduced the proliferation of SH-SY5Y cells in the dark (EC_50_ = 59 μM) but had no impact on cell growth after the cells had also been irradiated with green light.

**Table 2:** Cytotoxicity (EC_50_ with 95% Confidence Interval (CI), in μM) of [**1**](PF_6_)_2_–[**3**](PF_6_)_2_ in SH-SY5Y Cells Either Kept in the Dark or Following Green Light Irradiation (520 nm, 30 min, 25.2 J/cm^2^)^a^

compound	EC_50,D_ [μM]	CI_95_ [μM]	EC_50,GL_ [μM]	CI_95_ [μM]	PI
[**1**](PF_6_)_2_	>100		>100		
[**2**](PF_6_)_2_	>100		>100		
[**3**](PF_6_)_2_	34.2	+16.8	>100		
		–12.05			
donepezil HCl	59.1	+31.0	>100		
		–16.6			

a The cytotoxic experiments were performed under normoxic conditions (21% O_2_) in biological and technical triplicates; errors indicate 95% confidence intervals (CIs) in μM.

**Figure 4:**
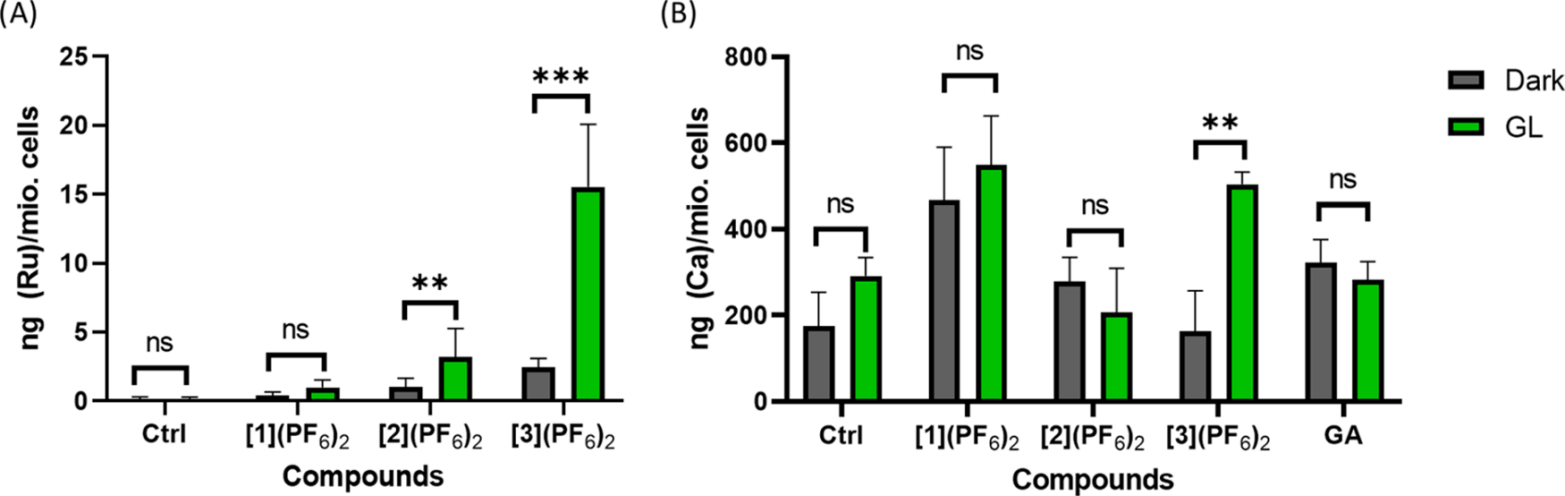
Ru (A) and Ca (B) content of SH-SY5Y cells treated with [**1**](PF_6_)_2_ and [**2**](PF_6_)_2_ (20 μM) and [**3**](PF_6_)_2_ (10 μM). Six hours after incubation, the cells were irradiated with green light (520 nm, 30 min 25.2 J/cm^2^) or kept in the dark. Untreated cells served as a negative control, while cells treated with l-glutamic acid (GA, 10 μM) were used as a positive control for enhanced Ca uptake. After an overall 30 h incubation time, the cells were collected, and Ca and Ru content was recorded via ICP-MS.

These surprising results led us to further investigate whether the uptake of the respective compounds plays a pivotal role in the alternation of the proliferation. First, we checked the ruthenium content of the most potent compound [**3**](PF_6_)_2_ (10 μM) in SH-SY5Y cells when cells were kept in the dark or irradiated with green light (520 nm, 30 min, 25.2 J/cm^2^). Therefore, we incubated SH-SY5Y cells with the complex, but instead of 24 h dark incubation time, we irradiated 6 h after compound addition, as previous studies on the U-87MG cell line did not show any significant difference in the Ru uptake level at 6 or 24 h incubation time. The complex was allowed to incubate for 24 h after irradiation before the ruthenium cellular content was analyzed by ICP-MS. As Figure 4A and Table S3 illustrate, a seven times higher Ru content was found after [**3**](PF_6_)_2_ had been activated with green light (15.5 ng (Ru)/ mio cells) compared to the unactivated complex (2.4 ng (Ru)/mio. cells) that is almost equal to that found in U-87MG cells. Like in U-87MG cancer cells, [**1**](PF_6_)_2_ (dark: 0.4 ng (Ru)/mio. cells; GL: 0.9 ng (Ru)/mio. cells) and [**2**](PF_6_)_2_ (dark: 1.0 ng (Ru)mio. cells; GL: 3.2 ng (Ru)/mio. cells) were poorly taken up even when twice the concentration (20 μM) was administered compared to [**3**](PF_6_)_2_. We finally decided to incubate the SH-SY5Y cells with the EC_50_ values obtained from the cytotoxicity experiment using the U-87MG cell, as those procedures would allow one to observe the effect of the prodrug on the healthy neuron-like cells when treated with the phototoxic compound dose. It is important to note that in all of the complexes we tested, the light-activated species resulted in higher concentrations of ruthenium within cells, suggesting that the activated drug may accumulate more effectively than the prodrug (as already observed in A549 and A375 cancer cells). For [**1**](PF_6_)_2_ and [**2**](PF_6_)_2_, these higher ruthenium levels after activation did not lead to a lower cell population, though. Overall, cellular uptake in neuron-like SH-SY5Y cells followed the same trends as those for GBM cells U-87MG in the dark, while uptake was much higher after light activation.

Another indication of the neurological toxicity of chemicals can be obtained by measuring the intracellular levels of calcium in healthy neurons. Ca^2+^ ions are important secondary messengers that regulate membrane excitability and dendrite development and contribute in various primary neuronal functions such as neurotransmitter synthesis and release, neuronal excitability, information processing, and memory storage.^72^ Dysregulation of intracellular Ca^2+^ ion concentration leads to neuronal cell death and brain damage, as demonstrated years ago in AD studies.^73−76^ Brain aging is also characterized by changes in calcium homeostasis.^77,78^

Thus, we investigated the impact of treatment with ruthenium complexes [**1**](PF_6_)_2_, [**2**](PF_6_)_2_, and [**3**](PF_6_)_2_ on the intracellular Ca^2+^ level of SH-SY5Y cells. Six hours after compound addition, the cells were irradiated with green light or kept in the dark. Intracellular Ca^2+^ levels were then assessed by ICP-MS 24 h after light activation. The NMDA and α-amino-3-hydroxy-5-methyl-4-isoxazole propionic acid (AMPA) receptors being glutamate-gated cation channels allow for an increase of calcium permeability of the neuron membrane; we also used l-glutamic acid (GA, 10 μM), a known NMDA and AMPA activator, as positive control in this assay. Glutamate showed twice higher Ca^2+^ levels (322 ng (Ca)/mio. cells) compared to vehicle control (175 ng (Ca)/mio. cells). As displayed in Figure 4B and Table S3, elevated Ca^2+^ levels were found for cells treated with [**1**](PF_6_)_2_ (467 ng (Ca)/mio. cells) and [**2**](PF_6_)_2_ (279 ng (Ca)/mio. cells) in the dark compared to untreated cells (175 ng (Ca)/mio. cells), while for [**3**](PF_6_)_2_, almost the same amount of calcium was found as in untreated cells (163 ng (Ca)/mio. cells). Strikingly, after light irradiation, the intracellular Ca^2+^ content increased 3-fold for activated [**3**](PF_6_)_2_ (504 ng (Ca)/mio. cells), while for [**1**](PF_6_)_2_ (549 ng (Ca) /mio. cells) and [**2**](PF_6_)_2_ (325 ng (Ca)/mio. cells), only a slight increase of Ca^2+^ was found compared to dark conditions. Notably, green light alone had a significant impact on intracellular Ca^2+^ accumulation, which increased 2-fold from 175 in the dark to 291 ng (Ca)/mio. cells in untreated, light-irradiated cells. Notwithstanding, we did not observe any correlation between elevated Ca^2+^ levels and reduced cell proliferation or the presence of floating cells within the treated wells.

Among all enzymes involved in transmembrane signaling, another important one is AChE. In a modified procedure (for further details, see the Experimental Section), we checked whether the ruthenium complexes were able to inhibit AChE in the dark and after light activation. The colorimetric assay used Ellman’s reagent. In short, [**1**](PF_6_)_2_, [**2**](PF_6_)_2_, and [**3**](PF_6_)_2_ were incubated at different concentrations with the isolated enzyme and kept in the dark or irradiated with green light (520 nm, 30 min, 25.2 J/cm^2^). The conversion of 5,5′-dithiobis(2-nitrobenzoic acid) (DTNB) to yellow 2-nitro-5-thiobenzoate (TNB) initiated by the addition of acetylthiocholine was then detected by UV–vis spectroscopy every 5 min for a total time of 35 min. Donepezil HCl, a known AChE inhibitor, was used as the positive control. Respective IC_50_ values were calculated from the inhibition values 35 min after DTNB addition (Table 3 and Figures S3 and S4, Supporting Information). In the dark, almost all compounds had an IC_50_ above the highest concentration (15 μM) used, except [**3**](PF_6_)_2_, which had an IC_50_ of 9.6 μM. Upon light irradiation, all compounds were able to inhibit ∼50% of the enzyme activity. Nevertheless, no complex reached inhibitory effects comparable to that of donepezil HCl that blocked AChE activity completely at the lowest concentration (1 μM). Overall, the ruthenium complexes seemed to weakly inhibit AChE in a light-dependent manner, but none of them were significant inhibitors, notably compared to donepezil HCl.

**Table 3:** AChE Effective Inhibitory Concentrations (IC_50_ with 95% Confidence Interval in μM) of Ruthenium Compounds [**1**](PF_6_)_2_–[**3**](PF_6_)_2_ in the Dark and after Green Light Irradiation (GL; 520 nm, 30 min, 25.2 J/cm^2^)

compound	IC_50,D_ [μM]	95% CI	IC_50,GL_ [μM]	95% CI	PI
[**1**](PF_6_)_2_	>15		6.74	+0.96	>2.2
				–0.92	
[**2**](PF_6_)_2_	>15		10.29	+3.88	>1.5
				–2.10	
[**3**](PF_6_)_2_	9.60	+3.26	5	+0.45	2
		–1.96		–0.44	
donepezil HCl	<1		<1		

### Computational Study

**Scheme 2:**
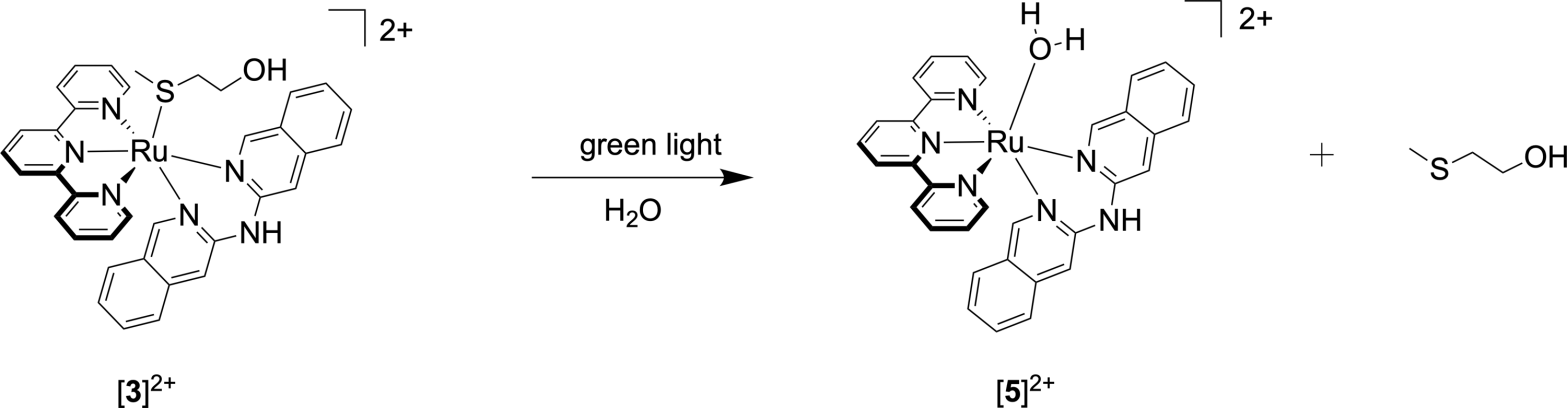
Chemical Structures Used for Docking Studies with MetalDock; the Aqua Ligand of [**5**]^2+^ was Removed from the x,y,z Coordinate File before Docking

The uptake of Ca^2+^ ions is regulated by not only a multitude of transport systems, e.g., the direct NMDA and AMPA receptors, but also indirectly via serotonin receptors and calcium/calmodulin-dependent protein kinase II and IV (CAMK2 and CAMK4). The substantial increase in intracellular Ca^2+^ levels after irradiation of [**3**](PF_6_)_2_ led us to wonder whether this metal complex may inhibit Ca^2+^ transporters. Having noticed a comparable trend in the cytotoxicity between [**3**](PF_6_)_2_ and donepezil HCl on the U-87MG and SH-SY5Y cell lines, we decided to perform a docking study of donepezil HCl to all of the calcium receptor targets. Additionally, we performed a computational screening of the inhibitory properties of the ruthenium compound [**3**]^2+^ and its light-activated aqua analogue [**5**]^2+^ (see Scheme 2) toward different calcium transmembrane transport systems to identify their potential inhibitory targets.

**Figure 5:**
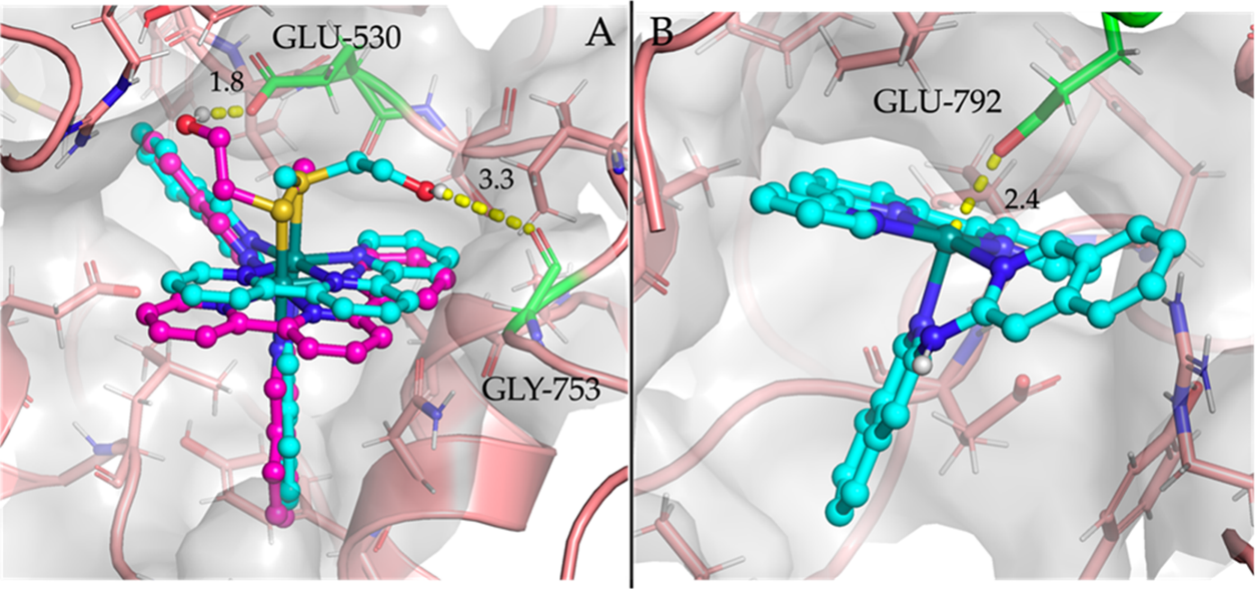
Poses obtained by docking [**3**]^2+^ (A) and [**5**]^2+^ (B) in the pocket of NMDA (PDB: 7EOR). The hydroxyl group of the Hmte ligand of [**3**]^2+^ forms a hydrogen bond with the carbonyl of the backbone of GLY-753 (cyan) or the carboxylate of GLU-530 (purple), whereas the ruthenium atom of [**5**]^2+^ is coordinated to the negatively charged carboxylate of GLU-792. Distances are given in Angstrom. Residues that form an interaction with the compounds are highlighted in green.

Using the MetalDock docking program recently published by our group,^79^ we investigated the interaction of the nonirradiated compound, [**3**]^2+^, where Hmte is still coordinated to ruthenium, and of the light-activated analogue, [**5**]^2+^, where Hmte is dissociated, and there is hence an aqua ligand bound to ruthenium instead. After photosubstitution of Hmte by water, coordination of metal-binding residues of the different protein targets to ruthenium is possible, which usually renders docking studies impossible. Our MetalDock program, however, allows testing of such metal-based inhibitors. The water molecule of [**5**]^2+^ was hence removed, and both ruthenium complexes were docked with NMDA (PDB: 7EOR), AMPA (PDB: 5YBG, 5ZG0, 4LZ5, 4LZ7), CAMK2 (PDB: 2VZ6), CAMK4 (PDB: 2VZ2), serotonin (7WC4, 7WC6), and AChE (PDB: 4EY7) receptors after removing the cocrystallized organic inhibitor usually present in published crystal structures. We used a 30 Å × 30 Å × 30 Å box centered around the original organic inhibitor bound to the protein X-ray structure in the PDB files. During each docking simulation, 10 poses were generated. Strikingly, the docking of [**3**]^2+^ and [**5**]^2+^ to the active site of the NMDA receptor resulted in poses that fitted very well in the binding pocket and showed clear-cut interactions with different residues. For [**3**]^2+^, four poses were obtained in which the Hmte ligand formed a hydrogen bond with the carboxylate of GLU-530, while four others showed the Hmte ligand forming a hydrogen bond with the carboxylate of GLY-753 (Figure 5A) and the remaining two poses displayed less prominent interactions. In all 10 poses of [**5**]^2+^, the ruthenium atom interacted with the negatively charged carboxylate of the GLU-792 residue (Figure 5B). Thus, [**3**]^+^ and [**5**]^2+^ can interact in the same manner as the allosteric inhibitor ifenprodil,^80^ pointing to the inhibitory effects of our Ru compounds, and the binding to the NMDA receptor is based on our experimental data unlikely. Two distinct crystal structures, namely, PDB 7WC6 and 7WC4, were utilized for the serotonin receptor. In 7WC6, no poses were identified that exhibited any notable interaction with the protein. Conversely, poses obtained after docking with 7WC4 revealed some interactions. 3 of the 10 poses of [**3**]^2+^ displayed no significant interaction, while three other poses exhibited a hydrogen bond interaction of the hydroxyl of the Hmte ligand with the backbone of PHE-234, and four poses established a similar interaction with the carboxylate group of ASP-155 (Figure S5A). For [**5**]^2+^, three poses were located outside the binding pocket, while the remaining seven were situated within the pocket but lacked coordination interactions with the ruthenium atom. Only a long-range hydrogen bond interaction of 3.5 Å with SER-219 was observed (Figure S5B). Those data suggest that [**3**]^2+^ might bind at similar amino acids as serotonin but not as LSD. In contrast to the NMDA and serotonin receptors, the docking procedure of [**3**]^2+^ and [**5**]^2+^ with receptors CAMK2, CAMK4, and AChE did not result in any pose exhibiting significant interaction with the protein. Overall, these results indicate that an interaction of [**3**]^2+^ and [**5**]^2+^ with the NMDA receptors is more likely than binding to serotonin receptors.

**Figure 6:**
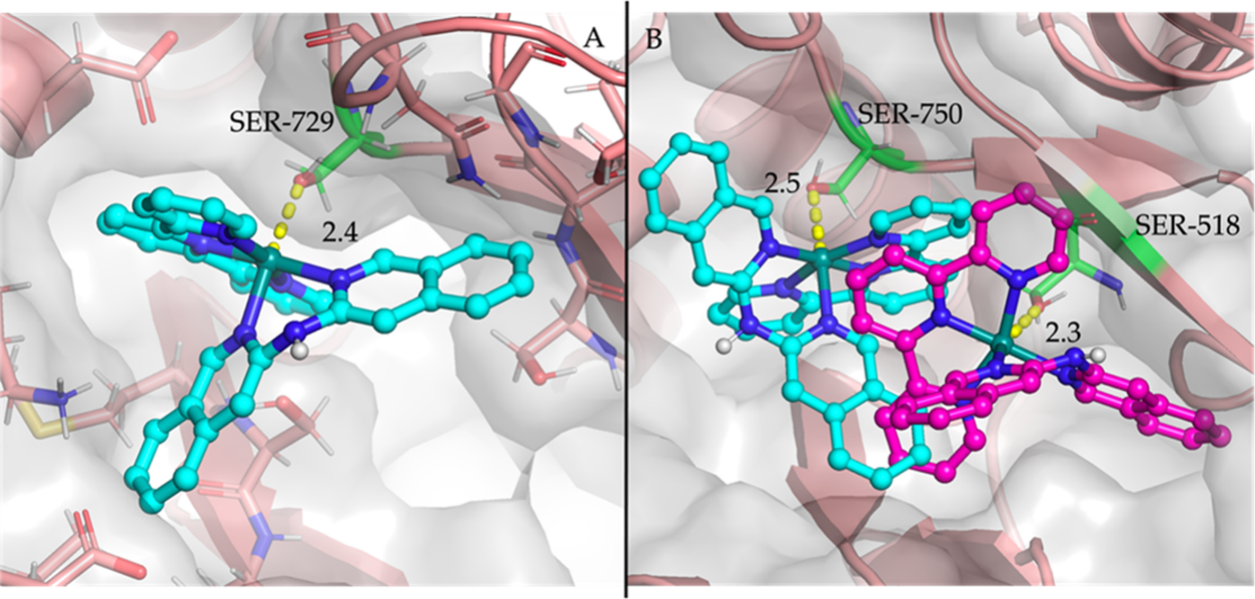
Interaction of [**5**]^2+^ and the AMPA receptor according to the MetalDock program. (A) Best pose of [**5**]^2+^ in the PDB 4LZ5 structure showing coordination to the SER-729 residue. (B) Two best poses of [**5**]^2+^ in the PDB 5YBG structure showing coordination to the SER-750 and SER-518 residues.

For the AMPA receptor, we decided to use three different PDB (4LZ5, 4LZ7, 5YBG) structures that included either the natural inhibitor glutamate or an allosteric inhibitor. For the PDB file 4LZ7, no interactions with [**3**]^2+^ or [**5**]^2+^ and the binding pocket were found. Also, for the other two structures, we found that [**3**]^2+^ did not form a strong coordination bond in a binding pocket. For [**5**]^2+^, the ruthenium atom was found to interact with the oxygen of the hydroxyl group of a serine residue within the active site (Figure 6). In PDB entry 4LZ5, all poses were found to interact with SER-518 (Figure 6A). The docking procedure of [**5**]^2+^ to 5YBG identified two possible sites of interaction, SER-518 and SER-750 (Figure 6B). The identification of the same interaction over these two different PDB structures highlights the high probability that [**5**]^2+^ interacts with a serine residue in the pocket of the AMPA receptor. This docking screening identified three possible calcium transmembrane transporter targets that [**3**]^2+^ and [**5**]^2+^ may potentially inhibit: NMDA, the serotonin receptor, and the AMPA receptor. There is a notable difference in the interaction strength of the obtained poses of [**3**]^2+^ and [**5**]^2+^, which could potentially explain the observed difference in Ca^2+^ concentration when the cells were irradiated with light or kept in the dark. Of course, future experimental investigations should confirm these initial computational results.

## General Discussion

Albeit ruthenium-based PACT compounds have already demonstrated their anticancer effect in a plethora of human cancer cell lines in vitro and in vivo, many ruthenium polypyridine photocages have been reported to be non-biologically active or poorly biologically active and act mostly as carriers for organic inhibitors.^50,81,82^ In addition, the use of ruthenium complexes to treat diseases that affect the brain has been sparsely used to date.^53^ In fact, only a few examples of anticancer^53^ or anti-AD^65−67^ ruthenium-based compounds have been published. Despite their effectiveness against cancer or in the resolubilization of Alzheimer’s proteins, little attention has been paid to their effects on the healthy neuronal network.

Within the series of structurally related compounds [**1**](PF_6_)_2_–[**3**](PF_6_)_2_, in this new work, several trends could be observed that were highly dependent on the nature of the bidentate ligand. As expected, [**1**](PF_6_)_2_ was poorly phototoxic due to very low uptake. Surprisingly, changing the bpy ligand by *i*-biq increased cytotoxicity at least by a 3-fold factor, although uptake did not increase significantly; still, no difference between dark and light cell growth inhibition EC_50_ values was seen for [**2**](PF_6_)_2_. The best light-activated properties in this series of compounds were observed for [**3**](PF_6_)_2_, which became 3.5 times more toxic upon green light irradiation compared with dark conditions. Compared with [**2**](PF_6_)_2_, it was twice less cytotoxic in the dark in spite of the 4 times higher uptake and twice more cytotoxic after light irradiation. Similar to our previous study, in A375 and A549 cells,^56^ the bidentate ligand regulates the ability of cells to take up these compounds, which increases in the order [**1**](PF_6_)_2_ < [**2**](PF_6_)_2_ < [**3**](PF_6_)_2_. The target of those kind of ruthenium-based compounds is still elusive; however, our observations indicate that [**2**](PF_6_)_2_ interacts with its biological target independently of the photoreleased ligand, whereas [**3**](PF_6_)_2_ showed more light-induced toxicity.

The antiproliferative effects of [**3**](PF_6_)_2_ in U-87MG cells made it a good candidate for more detailed neuro-safety investigations. Interestingly, light penetration had a pivotal impact on the growth of neuron-like SH-SY5Y cells as well as on Ru^2+^ uptake and, unexpectedly, on Ca^2+^ cellular uptake. Treatment with our complexes and green light even triggered the proliferation of this cell line or blocked the antiproliferative effect of green light alone. This strange effect was very similar to that observed, under the same conditions, with donepezil HCl, which is supposed to not be photoactive. Several studies pointed out that green light treatment alone can reduce pain^83,84^ or cause dendrite elongation, which is connected to increased connectivity and communications between neurons.^71^ Alon *et al.* found when irradiating SH-SY5Y with green light LED diodes that the cells showed enhanced neurite development, which is associated with neuronal repair.^71^ It is difficult to state if these observations can be attributed to the effects of green light or the drugs themselves, as complex [**1**](PF_6_)_2_ (slightly) reduced the proliferation after irradiation with green light. Despite high Ru concentrations being found in SH-SY5Y cells, these concentrations did not result in enhanced cytotoxicity. We can exclude that the increase in the dose–response curve in high concentrations is due to the absorbance of the complexes that might overlap with SRB (510 nm) and therefore result in a higher absorbance. If this were the case, the same results would have been expected in the experiment using U-87MG cells and in previous studies (see ref (56)). The cellular uptake studies revealed that upon light activation, two to eight times more Ru found within the cells as compared with dark conditions. Nevertheless, the increased proliferation effect upon green light activation cannot be reducible to the amount of Ru^2+^ ions inside the cell. For example, light-activated [**2**](PF_6_)_2_ showed an increased proliferation upon green light treatment, even though five times less Ru^2+^ was recorded in SH-SY5Y cells after light exposure. Consequently, no correlation could be drawn between ruthenium uptake and its inhibitory effects on cell proliferation in SH-SY5Y cells. It even appears that the Ru complex, in combination with green light, stimulates cell growth. The reason why we observed an increased proliferation upon green light treatment and higher concentrations of, in particular, [**2**](PF_6_)_2_ is still elusive and requires further investigations such as proteomic studies or protein level expression upon drug and light treatment.

The use of green light also had an effect on Ca^2+^ uptake in the control cells that were not treated with Ru-based drugs. Under resting conditions, or not excited conditions, the intracellular Ca^2+^ levels in neurons is approximately 0.1–0.5 μM.^72^ If those levels are below or above, it can cause fatal consequences, also known as excitotoxicity. Excitotoxicity can lead to neurodegeneration, loss of memory, and, in fatal cases, stroke. High levels of Ca^2+^ are associated with irreversible cell damage.^68,75^ Crucial processes such as proliferation and cancer progression are Ca^2+^-dependent.^85−87^ When we treated the control group with green light, we observed that the intracellular Ca^2+^ amount increased to twice the amount when we left the control cells in the dark. As expected, GA treatment in the dark triggered Ca^2+^ uptake to twice the amount compared with that found in the control cells in the dark. Exceptional results were found by the light treatment of [**3**](PF_6_)_2_. Almost three times more Ca^2+^ was found when the cells were exposed to the PACT agent and green light. It is noteworthy that such a correlation between Ru^2+^ and Ca^2+^ uptake has not yet been reported. Some ruthenium amine complexes have been reported to inhibit mitochondrial calcium uptake via the blockage of the mitochondrial calcium uniporter.^88,89^ To the best of our knowledge, a sharp increase in Ca^2+^ levels following treatment with Ru-based compounds has not been previously observed. The enhanced proliferation rate ([**2**](PF_6_)_2_) or the reduced antiproliferative effect ([**1**](PF_6_)_2_ and [**3**](PF_6_)_2_), respectively, did not correlate with increased Ca^2+^ levels upon green light activation. Although high Ca^2+^ levels might suggest excitotoxicity, our experimental data do not support the hypothesis that our Ru-based PACT compounds cause cell death due to elevated Ca^2+^. In fact, the data suggest the opposite: our compounds might have cell-protective properties.

Of course, the effect of a ruthenium-based PACT compound on calcium cellular uptake opens many questions. Our docking studies suggested various potential mechanisms that may trigger such an increase in Ca^2+^ accumulation, namely, NMDA, serotonin, and/or AMPA receptor activation. Docking does not model electrons explicitly, and a real covalent interaction cannot be formed in our computational approach. In addition, ruthenium(II) is a soft atom, and oxygen-based ligands are often seen as bad ligands for this type of ion. Nonetheless, docking indicates clearly a good fit between the binding pocket and the shape of the molecule, and it is not impossible that a covalent interaction may form between ruthenium in the activated molecule and the oxygen atom of the serine residue of the AMPA receptor. Such interaction may explain the sharp increase in Ca^2+^ uptake upon treatment with [**3**](PF_6_)_2_ and green light irradiation. As noted, it is important to distinguish between extrasynaptic and synaptic AMPA receptors, as both play a controversial role. Our investigation revealed interaction hits across all of the docking receptors. However, in contrast to [**3**]^2+^ and [**5**]^2+^, distinct interactions were observed for NMDA and AMPA receptors. Although [**3**]^2+^ displayed a comparable interaction involving van der Waals and hydrogen bonding near the pocket where Donepezil HCl would interact, [**5**]^2+^ primarily demonstrated a single, strong coordination interaction of the ruthenium atom with the GLU-792 residue. Donepezil HCl, on the other hand, showcased a superior fit within the binding pocket (Figure S6), leading to a more potent van der Waals interaction. While this alternative mode of interaction could still yield similar effects, a conclusive understanding would necessitate further exploration of these binding modes through more advanced models such as molecular dynamics.

Nevertheless, AMPA activation is associated with drugs causing seizures.^90^ Also, the stimulation of extrasynaptic NMDA receptors are reported to contribute to cell death,^91^ while evidence suggested that the activation of synaptic NMDA receptors play a role in the longevity and health of cells.^92^

AChE is also a complex receptor. Several studies have demonstrated that AChE activity is associated with cancer cell proliferation,^93−95^ some cancers overexpress the AChE enzyme, resulting in uncontrolled cell growth and drug resistance,^95^ and apoptotic cells show increased AChE activity.^96^ Although the ruthenium complexes tested in this study showed light-dependent inhibitory effects in a chemical assay using the isolated AChE enzyme, these data must be considered with care. It is difficult to draw conclusions on the intracellular behavior of drugs from such a chemical enzymatic study. For example, donepezil HCl inhibited AChE activity at nM concentrations in our chemical assay, but in cells, it showed no significant effect on cell proliferation, neither in U-87MG nor in SH-SY5Y cells (Tables 1, 2 and Figure S2). In order to investigate if AChE inhibition really plays a role, further investigations are required, such as different expression levels of AChE in both cell lines and cell-based AChE inhibition studies.^97^

## Conclusions

In this work, we have described the cytotoxic effects of different Ru polypyridine complexes in U-87MG glioblastoma cells. While in vitro efficacy studies for glioblastoma treatments come with inherent limitations, our findings strongly suggest the potential of ruthenium-based photoactivated chemotherapy (PACT) complexes such as [**3**](PF_6_)_2_ for further exploration in glioblastoma therapy. Prodrug [**2**](PF_6_)_2_ bearing the *i*-biq ligand already reduced proliferation of GBM cells in the dark, but it showed no activation by light, while [**3**](PF_6_)_2_ had limited growth inhibitory effect in the dark but was efficiently activated by green light (PI = 3.5). For the first time, we also examined the biological activity of these compounds in neuron-like SH-SY5Y cells as the first step toward safety assessment. Our results indicate that these compounds are cytotoxic only at high concentrations in these noncancerous cells. Notably, [**3**](PF_6_)_2_ showed a surprising reversal of toxicity upon light irradiation, a phenomenon similarly observed with cholinesterase inhibitor donepezil HCl. Importantly, no correlation was found between Ru uptake in SH-SY5Y cells and reduced cell proliferation. However, clear-cut effects of these ruthenium compounds were observed for the first time on calcium cellular uptake. We found a structure-dependent, light-dependent, and uptake-independent increase in Ca^2+^ uptake of cells treated with [**3**](PF_6_)_2_. Computational docking studies suggested that light-activated [**3**]^2+^ (i.e., [**5**]^2+^) but not [**3**]^2+^ may interact with the NMDA receptor, similar to the case for the known NMDA inhibitor ifenprodil. As we observed higher instead of lower Ca^2+^ levels, the binding to the NMDA receptor is unlikely within SH-SY5Y cells. Our docking results suggest that the AMPA receptors may be activated by [**5**]^2+^, which would explain elevated Ca^2+^ levels. Our cell studies highlighted an intriguing difference between U-87MG and SH-SY5Y cells: upon treatment with [**3**](PF_6_)_2_, light activation increases the toxicity in U-87MG cells, while the toxicity in SH-SY5Y cells decreases. This differential response emphasizes the need to consider both cancer and neuron-like cellular models when evaluating the safety and efficacy of Ru-based PACT compounds. Overall, our study suggested that Ru-based PACT compounds have promising potential for the treatment of GBM but that neurotoxicity and excitotoxicity must be investigated in more details. These considerations have, to our knowledge, not yet been considered, while they should remain central in the design of new Ru PACT compounds for the treatment of glioblastoma to avoid unintended interactions with Ca^2+^ transporters. Overall, studying these interactions experimentally and using more advanced computational methods to model them will be critical not only to better understand how ruthenium compounds behave in the brain and influence calcium effluxes but also as essential steps toward more translational approaches for the treatment of brain cancer with PACT.

## Experimental Section

### General Materials and Methods

Chemical reagents and solvents were purchased from commercial suppliers (Sigma-Aldrich, Fluka, Alfa Aesar, and Acros) and were used without further purification. Compounds [**1**](PF_6_)_2_–[**3**](PF_6_)_2_ were obtained as described elsewhere.^56^ Donezepil HCl was obtained from Sigma-Aldrich (D6821).

### Stability in the Presence of Glutathione

UV–vis spectroscopy experiments were performed on a Cary 60 Varian spectrometer equipped with a temperature control set to 310 K and a magnetic stirrer. The measurements were performed in a quartz cuvette containing 3 mL of solution. Complexes [**1**](PF_6_)_2_ (*c* = 0.05 mM), [**2**](PF_6_)_2_, and [**3**](PF_6_)_2_ (*c* = 0.08 mM) was dissolved in deionized water. Then, the UV–vis spectrum was recorded without the presence of GSH. After 4 min, GSH was added (0.5 mM) and UV–vis spectra were recorded directly after and after 2 and 24 h, while the vial was kept in the dark and at 37 °C.

### General Cell Culture Methods

The glioblastoma U-87MG cells were purchased from ATCC (American Type Culture Collection, Manassas, Virginia), and the neuroblastoma SH-SY5Y cells were kindly provided by the Department for Molecular Physiology, Leiden University, The Netherlands. Cells were grown in Dulbecco’s modified Eagle medium (DMEM) with phenol red (U-87MG) or DMEM/F12 with phenol red (SH-SY5Y, Sigma-Aldrich), supplemented with l-glutamine (1%), penicillin and streptomycin (0.1%), and fetal calf serum (FCS, 10%) at 37 °C in a 5% CO_2_/95% air atmosphere and fed/passaged twice weekly.

Dulbecco’s modified Eagle medium (DMEM, without phenol red, without glutamine), DMEM/F12, glutamine-S (GM; 200 mM), trichloroacetic acid (TCA), glacial acetic acid, sulforhodamine B (SRB), and tris(hydroxylmethyl)aminomethane (Tris base) were purchased from Sigma-Aldrich. FCS was purchased from Hyclone. Penicillin and streptomycin were purchased from Duchefa and were diluted to a 100 mg/mL penicillin/streptomycin solution (P/S). Trypsin and OptiMEM (without phenol red) were purchased from Gibco Life Technologies. Trypan blue (0.4% in 0.81% sodium chloride and 0.06% potassium phosphate dibasic solution) was purchased from BioRad. Plastic disposable flasks and 96-well plates for cytotoxicity assays were purchased from Sarstedt (No. 83.3924). Cells were counted using a BioRad TC20 automated cell counter with Biorad cell-counting slides (No. 1450015). Cells were inspected with an Olympus IX81 microscope. UV–vis measurements for the analysis of 96-well plates were performed with a M1000 Tecan Reader, Tecan Trading AG, Switzerland; plate shaking was performed on a GFL 3016 reciprocating horizontal shaker, Gesellschaft für Labortechnik, Germany.

### Cytotoxicity in U-87MG and SH-SY5Y Cells

For each photocytotoxicity experiment, two plates were prepared and treated identically except for the light irradiation part^56,60,82^ to test the cytotoxicity in the dark and following light activation. U-87MG and SH-SY5Y cells were seeded at *t* = 0 h in 96-well plates at densities of 6.000 and 8.000 cells/well (100 μL), separately, in OptiMEM supplemented with 2.5% v/v FCS, 0.1% v/v P/S, and 1.0% v/v GM (hereafter called OptiMEM complete) and incubated for 24 h at 37 °C and 5.0% CO_2_. Only the inner 60 wells were used for seeding, and the outer wells were equipped with 100 μL of OptiMEM to prevent border effects during irradiation. At *t* = 24 h, aliquots (100 μL) of six different concentrations (1, 10, 25, 50, 75, and 100 μM) of freshly prepared stock solutions (10 mM) of the compounds in OptiMEM complete were added to the wells in triplicate and incubated for an additional 24 h. Sterilized dimethyl sulfoxide (DMSO) was used to dissolve the compounds in such amounts that the maximum v/v% of DMSO per well did not exceed 0.5 vol % at the highest compound concentration. At *t* = 48 h, the light-irradiated plates were irradiated with our published cell-irradiation setup^98^ (520 nm, 30 min, 25.2 J/cm^2^) without changing the medium, while the control dark plates were kept in the dark. After irradiation, all plates were further incubated in the dark for an additional 48 h. The cells were fixed at *t* = 96 h by adding cold TCA (10% w/v; 100 μL) in each well, and the plates were stored at 4 °C for 24 h. The TCA medium mixture was removed from the wells and rinsed with demineralized water (3×). Afterward, each well was stained with 100 μL of SRB (0.6% w/v in 1% v/v acetic acid) for 30 min shaking with a GFL 3016 reciprocating horizontal shaker at 0–300 rpm. The SRB solution was removed by washing with acetic acid (1% v/v) and air-dried. The SRB dye was solubilized with Tris base (10 mM; 200 μL) overnight, and the absorbance in each well was read at λ = 510 nm using an M100 Tecan Reader.

The SRB absorbance data for each compound and concentration were averaged over three identical wells (technical replicates, *nt* = 3) in Excel and were exported to GraphPad Prism 9.0 (San Diego, CA). Relative cell populations were calculated by dividing the average absorbance of the treated wells by the average absorbance of the untreated wells. It was checked that the cell viability of the untreated cells of the samples irradiated was similar (maximum difference of 10%) to that of the nonirradiated samples to make sure no harm was done by light alone. The resulting dose–response curve for each compound in dark and irradiated conditions was fitted to a nonlinear regression function with fixed *y* maximum (100%) and minimum (0%, relative cell viability) and a variable Hill slope (eq 1).
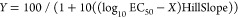
1

The data of three independent biological replications were averaged to obtain the final effective concentrations (EC_50_ in μM). Photo indices (PIs) were calculated for each compound by dividing the EC_50_ value obtained in the dark by the EC_50_ value determined under light irradiation.

### Cellular Uptake Studies in U-87MG Cells

Materials: 65% nitric acid (Suprapur, Merck) was used in the sample digestion process, while diluted 1% nitric acid (v/v) was employed as a carrying solution throughout the ICP measurements. For preparation of calibration and internal standards, National Institute of Standards and Technology (NIST)-traceable 1000 mg/L elemental standards were used (TraceCERT, Fluka). Approximately 18 MΩ cm^–1^ water (Milli-Q) was employed in all sample preparation and analysis steps.

Instrumentation: Calibration standards were prepared in a Secuflow fume hood (SCALA) to prevent contamination by atmospheric particulates. The standard samples and measurement samples were analyzed for trace elements using the NexION 2000 (PerkinElmer) ICP-MS instrument equipped with a concentric glass nebulizer and a Peltier-cooled glass spray chamber. An SC2 DX autosampler (PerkinElmer) was connected to ICP-MS for sample introduction. Syngistix software for ICP-MS (v.2.5, PerkinElmer) was used for all data recording and processing. Five trace elemental calibration standards for ICP-MS analysis were prepared using NIST-traceable 1000 mg/L Ru standards: 0, 1, 5, 20, and 100 μg/L. Samples were analyzed without dilution in the original delivery containers to minimize the possibility of contamination. Here, 10 μg/L Rh and In were used as internal standards. To check the calibration, samples were analyzed with a blank measurement and a repeat measurement with one of the calibration standards. For the calibration curve, the accepted correlation coefficient (Cor···Coeff) was found to be higher than 0.999.

Sample preparation: 1 mL of U-87MG cells were seeded in 12-well plates (Greiner, No. 665180; 400.000 cells per well). After 24 h, 10 μM of complexes [**1**](PF_6_)_2_, [**2**](PF_6_)_2_, and [**3**](PF_6_)_2_ were added and the mixture was incubated for 6 or 24 h separately. Afterward, the cells were trypsined, collected, and centrifuged. The resulting cell pellet was dissolved in 1 mL of PBS and counted via a BioRad TC20 automated cell counter. Afterward, the solution was centrifuged and washed again with PBS with subsequent centrifuging. This washing step was repeated twice. The resulting cell pellet was digested in 0.5 mL of 65% HNO_3_ overnight in a hot oven (90 °C). The solution was diluted with Milli-Q water to 10 mL, and the Ru content [ppb] was measured by ICP-MS.

### Cell Fractioning

Ru content in different compartments was determined via a FractionPREP Cell Fractionation Kit (Abcam, Cambridge, U.K., no 288085). U-87MG cells were seeded in 25 cm^2^ OptiMEM medium (1.5 × 10^6^ cells) for 24 h. Then, 10 μM [**2**](PF_6_)_2_ and [**3**](PF_6_)_2_ were added in three adjacent wells (technical triplicates). Untreated cells served as control. After 24 h of incubation, the cells were collected, counted, and fractioned according to the manufacturer’s procedure. In short, the cells were collected and centrifuged at 700*g* for 5 min. The cells were then washed with ice-cold PBS (1 mL) followed by 5 min centrifuging at 700*g*, and the supernatant was removed. The cell pellet was resuspended in 1 mL of ice-cold PBS, transferred into a 1.5 mL Eppendorf tube, and spun for 5 min at 700*g*, and the supernatant was removed. The pellet was resuspended in 400 μL of Extraction Buffer III/Cytosol Extraction Buffer Mix [containing 2 μL of dithiothreitol (DTT) and 2 μL of protease inhibitor cocktail per mL]. The cells were mixed well by carefully pipetting up and down (ca. 5 times). The sample was incubated on ice for 20 min with gentle tapping three to four times every 5 min. Afterward, the sample was centrifuged at 700*g* for 10 min. The supernatant was collected (cytosolic fraction) and kept on ice. The resulting pellet was again resuspended in 400 μL of ice-cold Extraction Buffer IV/Membrane Extraction Buffer-A Mix (containing 2 μL of DTT and 2 μL of protease inhibitor cocktail per mL) and pipetted several times, and the sample was vortexed for 10–15 s to mix well. Then, 22 μL of Lysis Buffer/Membrane Extraction Buffer-B was added and vortexed for 5 s. The samples were incubated for 1 min on ice. Afterward, the sample was vortexed again for 5 s and centrifuged for 5 min at 1000*g*. The supernatant was immediately transferred to a clean, prechilled 1.5 mL tube and kept on ice (membrane fraction). The pellet was again resuspended in 200 μL of ice-cold Extraction Buffer V/Nuclear Extraction Buffer mix (containing 2 μL of DTT and 2 μL of protease inhibitor cocktail per mL), vortexed for 15 s, and kept on ice for 40 min with constant vortex for 15 s every 10 min. The sample was then microcentrifuged at top speed for 10 min. The resulting supernatant was transferred in a clean, prechilled 1.5 mL tube (nuclear fraction) and kept on ice. The remaining cytoskeletal fraction was dissolved in 100 μL of 0.2% SDS containing 10 mM DTT solution. All samples were digested over 65% HNO_3_ (0.5 mL) in an oven hot oven (90 °C). The solution was diluted with Milli-Q water to 10 mL, and the Ru content [ppb] was measured by ICP-MS.

### Cellular Uptake Studies in SH-SH5Y Cells

1 mL of SH-SY5Y cells were seeded in 12-well plates (Greiner, No. 665180; 500.000 cells per well). After 24 h, 20 μM complexes [**1**](PF_6_)_2_ and [**2**](PF_6_)_2_ and 10 μM [**3**](PF_6_)_2_ were added and incubated for 6 h. One plate was exposed to green light (520 nm, 30 min, 25.2 J/cm^2^), while the other one was kept in the dark. After an additional 24 h of incubation, the cells were trypsined, collected, and centrifuged. The resulting cell pellet was dissolved in 1 mL of PBS and counted via a BioRad TC20 automated cell counter. Afterward, the solution was centrifuged and washed again with PBS with subsequent centrifuging two more times. The resulting cell pellet was digested in 0.5 mL of HNO_3_ overnight in a hot oven (90 °C). The solution was diluted with Milli-Q water to 10 mL, and the Ru and Ca content [ppb] was measured via ICP-MS.

### Acetylcholine Esterase Inhibition Assay

An acetylcholine esterase inhibition kit was purchased from Abcam, Cambridge, U.K. (No. 138871) and was used with some modifications: The obtained enzyme was first dissolved in 100 μL of Milli-Q water with 0.1% bovine serum albumin to make a 50 units/mL solution. The solution was further diluted 1:50 in 50 mM Tris buffer, pH = 8 (10 μL in 490 μL), and then placed in 4.5 mL of 50 mM Tris buffer, pH = 8. The complexes were first dissolved in DMSO to obtain a 10 mM solution and then further diluted in 50 mM Tris buffer, pH = 8, to obtain 1, 2.5, 5, 7.5, 10, and 15 μM solutions. 50 μL of the enzyme and 50 μL of the complex solution were added to a 96-well plate in triplicates. Untreated enzymes served as the negative control, while treatment with donepezil HCl at the same concentrations (1, 2.5, 5, 7.5, 10, and 15 μM) as the complex solution was used as the positive control. The plates were kept for 30 min in the dark or exposed to green light irradiation (520 nm, 30 min, 25.2 J/cm^2^). Meanwhile, a 10 mM solution of acetylthiocholine iodide and 10 mM DTNB was prepared. After the respective time, in each well, 5 μL of the acetylthiocholine iodide and DTNB mixture was added. The absorbance was read at a microplate Tecan reader at 410 nm every 5 min for in total 40 min.

### Computational Studies

Docking studies were performed using MetalDock^79^ with the AMS 2021 engine^99^ at the TZP/PBE0/COSMO level,^100−103^ including Grimme’s D3 dispersion corrections with BJ damping.^104^ Relativistic effects were scalarly corrected for by ZORA.^105^ The box was centered on the molecule bound to the protein in the PDB files, and a 30 Å × 30 Å × 30 Å box was used. After deleting the atoms of the bound water molecule of [**5**]^2+^, we performed our docking simulations for [**3**]^2+^ and [**5**-H_2_O]^2+^, generating in total 10 poses. To analyze the docking results, we used PyMOL.^106^

## Supporting Information Available

UV–vis spectra of [**1**]–[**3**](PF_6_)_2_ in water in the presence of l-glutathione; dose–response curves in U-87MG of [**1**](PF_6_)_2_–[**3**](PF_6_)_2_ and donepezil HCl; tables of the Ru cellular uptake studies in U-87MG cells after 6 and 24 h; cellular fractioning of [**2**](PF_6_)_2_ and [**3**](PF_6_)_2_ in U-87MG cells; Ru and Ca uptake of [**1**](PF_6_)_2_–[**3**](PF_6_)_2_ in SH-SY5Y cells; dose–response curves of [**1**](PF_6_)_2_–[**3**](PF_6_)_2_ on the isolated AChE enzyme; and docking data of [**3**]^2+^ and [**5**]^2+^ in the serotonin receptor (PDF)
X,Y,Z coordinates of the 10 best poses of the docked structures shown in Figures S5 and S6 (ZIP)

## Abbreviations

5-ALA: 5-aminolevulinic acid
AChE: acetylcholine esterase
AMPA: glutamatergic α-amino-3-hydroxy-5-methyl-4-isoxazole propionic acid
BBB: blood–brain barrier
bpy: 2,2′-bipyridine
CAM2K: calcium/calmodulin-dependent protein kinase II
CAM4K: calcium/calmodulin-dependent protein kinase IV
CI: confidential index
DMEM: Dulbecco’s modified Eagle medium
DTNB: 5,5′-dithiobis(2-nitrobenzoic acid)
EC_50_: half-maximal effective concentration
FBS: fetal bovine serum
GABA: γ-aminobutyric acid
GBM: glioblastoma multiforme
GSH: L-glutathione
Hmte: 2-methylthioethanol
IC_50_: half-maximal inhibitory concentration
ICP-MS: inductively coupled plasma-mass spectrometry
*i*-biq: bisisoquinoline
*i*-diqa: di(isoquinolin-3-yl)amine
^3^MC: triplet metal centered
^3^MLCT: metal-to-ligand charge transfer excited state
^1^O_2_: singlet oxygen
PACT: photoactivated chemotherapy
PDT: photodynamic therapy
PI: photo index
PP IX: protoporphyrin IX
ROS: reactive oxygen species
SRB: sulforhodamine B
tpy: 2,2′;6′,2″-terpyridine
TNB: 2-nitro-5-thiobenzoic acid
