# Supplementary material for: Toward the Treatment of Glioblastoma Tumors Using Photoactivated Chemotherapy: In Vitro Evaluation of Efficacy and Safety
Source: ACS Pharmacol Transl Sci. 2025 Jan 30;8(2):484–98. doi: 10.1021/acsptsci.4c00600 (PMC11833736; doi:10.1021/acsptsci.4c00600)
Supplement: Supplementary file 1 — pt4c00600_si_001.pdf [file pt4c00600_si_001.pdf]

Supporting Information

Towards treatment of glioblastoma tumors using photoactivated chemotherapy: in vitro evaluation of efficacy and safety

*Sina Katharina Goetzfried, Matthijs L. A. Hakkennes, Anja Busemann, Sylvestre Bonnet**

Leiden Institute of Chemistry, Leiden University, Einsteinweg 55, 2333CC Leiden, The Netherlands
*Corresponding author: bonnet@chem.leidenuniv.nl

Contents

1. **Stability in the presence of nucleophiles** 2
2. **Cytotoxicity in U-87MG and SH-SY5Y cells** 3
3. **Cellular uptake studies** 4
4. **Cell Fractioning** 5
5. **Cellular uptake studies in SH-SY5Y cells** 6
6. **Acetylcholine esterase inhibition assay** 7
6. **Computational Section** 9
7. **References** 9

1. Stability in the presence of nucleophiles

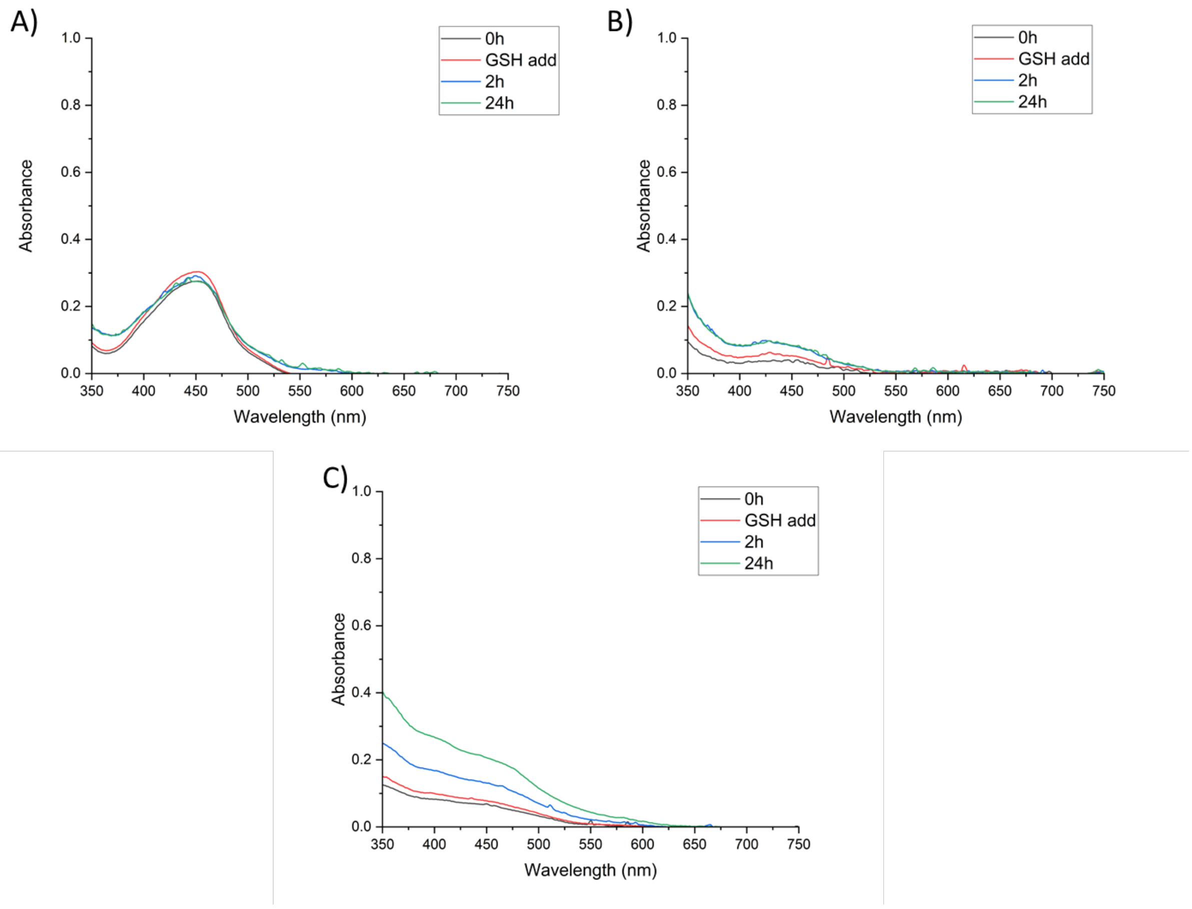

Figure S1. Evolution of UV-Vis spectra of a: [**1**]$(PF_6)_2$ (c = 0.05 mM), b: [**2**]$(PF_6)_2$ (c = 0.08 mM) and c: [**3**]$(PF_6)_2$ in deionized water (V = 3 mL, under air condition, T =). The UV-Vis spectra were measured immediately after dissolving, and after 4 min *L*-glutathione was added, further spectra were recorded after 2 and 24h in the dark.

2. Cytotoxicity in U-87MG and SH-SY5Y cells

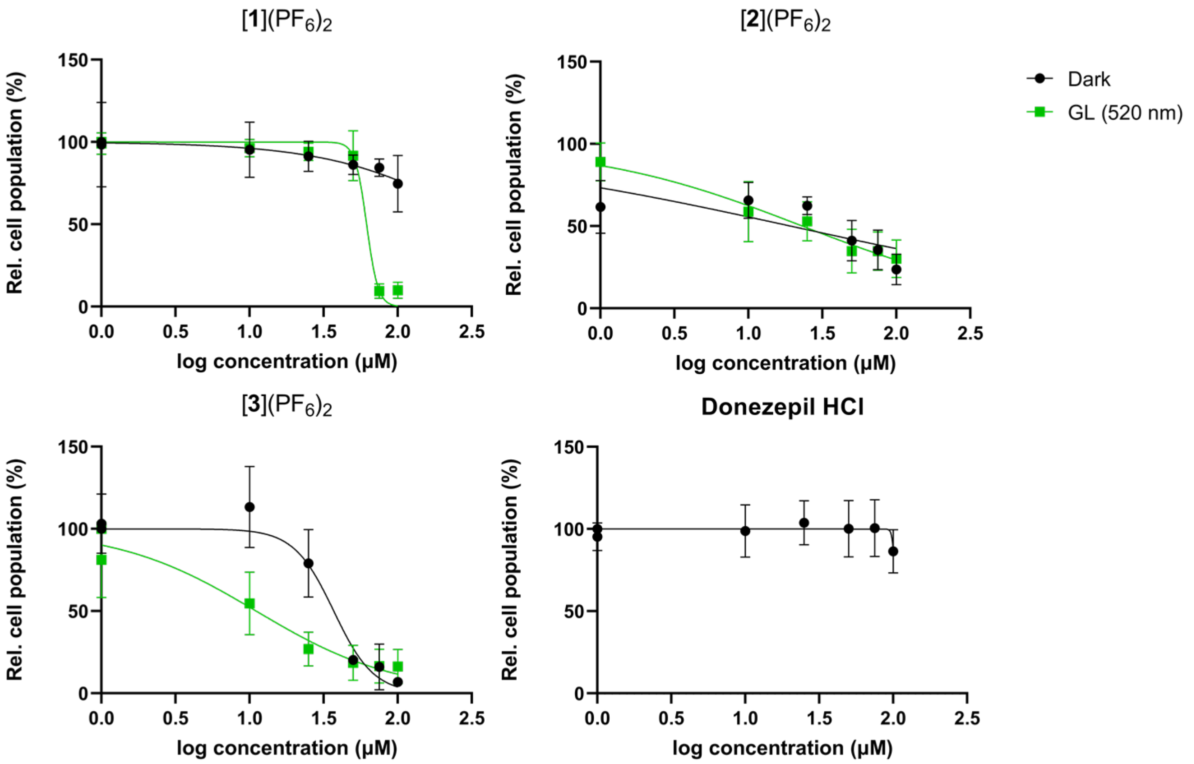

Figure S2. Dose-response curves in U-87MG cells treated with Donezepil (HCl salt), [**1**]$(PF_6)_2$-[**3**]$(PF_6)_2$ complexes in the dark (72 h) or incubated for 24 h, irradiated with green light (520 nm, 25.2 J/cm^2) and further incubated for 48 h.

3. Cellular uptake studies

Table S1. Ruthenium cellular uptake for [**1**]$(PF_6)_2$, [**2**]$(PF_6)_2$ and [**3**]$(PF_6)_2$ in ng Ru/mio. cells in U-87 MG cells after 6 or 24 h incubation time, respectively.

**compound**	**6 h**		**24 h**	
	ng Ru/mio. cells	Uptake in %	ng Ru/mio. cells	Uptake in %
**[1]$(PF_6)_2$**	0.60 ± 0.38	0.56	0.42 ± 0.11	0.43
**[2]$(PF_6)_2$**	0.54 ± 0.08	0.82	0.82 ± 0.09	1.5
**[3]$(PF_6)_2$**	2.14 ± 0.49	12.4	1.98 ± 0.33	11.5

4. Cell Fractioning

Table S2. Ruthenium cellular uptake for [**2**]$(PF_6)_2$ and [**3**]$(PF_6)_2$ in ng Ru/mio. cells in U-87MG cells in different cell compartments after 24 h incubation time.

compound	Cytosol	Membrane	Nucleus	Cytoskeleton
	ng Ru/mio. cells	ng Ru/mio. cells	ng Ru/mio. cells	ng Ru/mio. cells
**Untreated cells**	0.01 ± 0.02	0.0 ± 0.001	0.01 ± 0.006	0.0 ± 0.0003
**[2]$(PF_6)_2$**	0.18 ± 0.06	0.83± 0.57	0.03 ± 0.03	0.02 ± 0.01
**[3]$(PF_6)_2$**	0.07 ± 0.05	0.88 ± 0.25	0.17 ± 0.06	0.06 ± 0.03

5. Cellular uptake studies in SH-SY5Y cells

Table S3. Calcium and Ruthenium uptake in ng metal/mio. cells for [**1**]$(PF_6)_2$, [**2**]$(PF_6)_2$ and [**3**]$(PF_6)_2$ incubated with 20 or 10 µM, respectively, in SH-SY5Y cells. Cell were irradiated after 6 h incubation time with green light (520 nm, 30 min, 25.2 J/cm^2) or kept in the dark and collected after an overall incubation time of 24 h. Data represents average and standard deviation of three replicates.

	**Ca uptake**		**Ru uptake**	
	Dark	Green light	Dark	Green light
	ng Ca/mio. cells	ng Ca/mio. cells	ng Ru/mio. cells	ng Ru/mio. cells
**Untreated cells**	175.40 ± 78	291.54 ± 42	0.1 ± 0.02	0.01 ± 0.16
**[1]$(PF_6)_2$**	467.78 ± 122	549.39 ± 113	0.40 ± 0.25	0.98 ± 0.53
**[2]$(PF_6)_2$**	279.12 ± 56	325.75 ± 111	1.02 ± 0.63	3.19 ± 2.09
**[3]$(PF_6)_2$**	163.37 ± 93	504.02 ± 29.2	2.44 ± 0.63	15.52 ± 4.54
**Glutamic acid**	322.84± 53	282.89 ± 42	--	-

6. Acetylcholine esterase inhibition assay

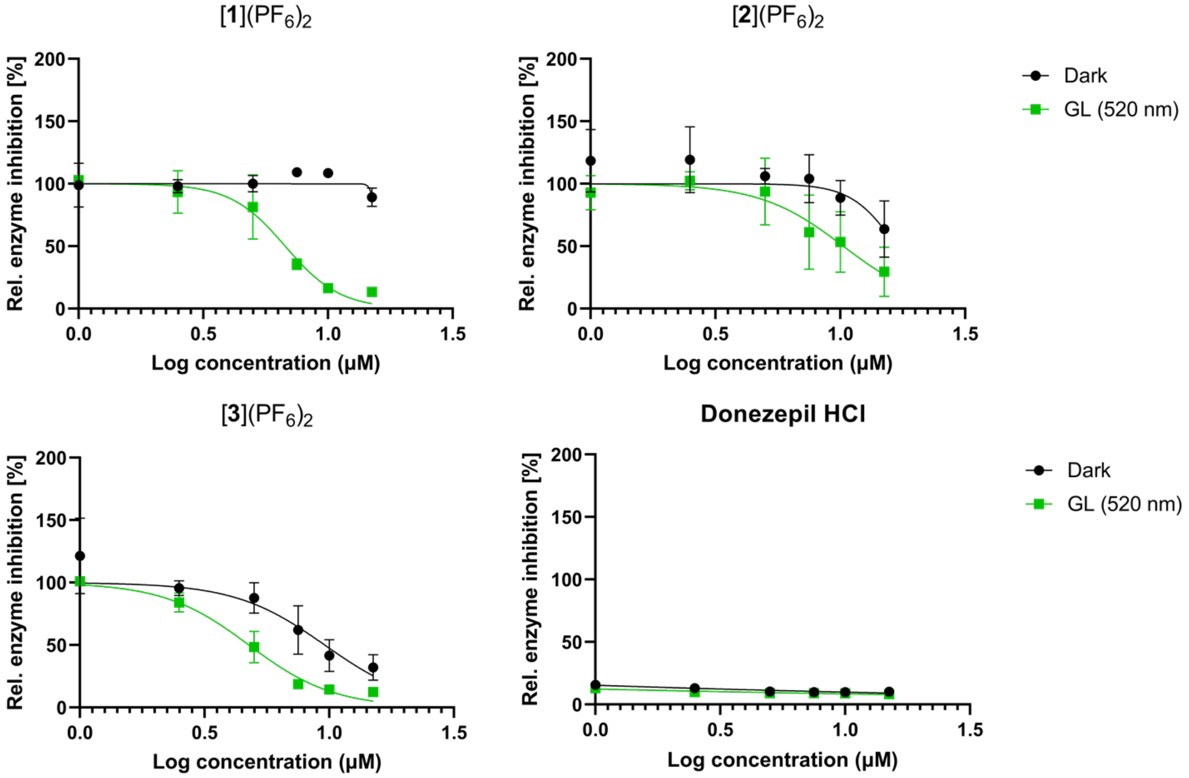

Figure S3. Dose-response curve of the enzymatic activity of isolated AChE treated with different concentrations of Donezepil (HCl salt), [**1**](PF_6)$_2$-[**3**](PF_6)$_2$ complexes after 30 min incubation.

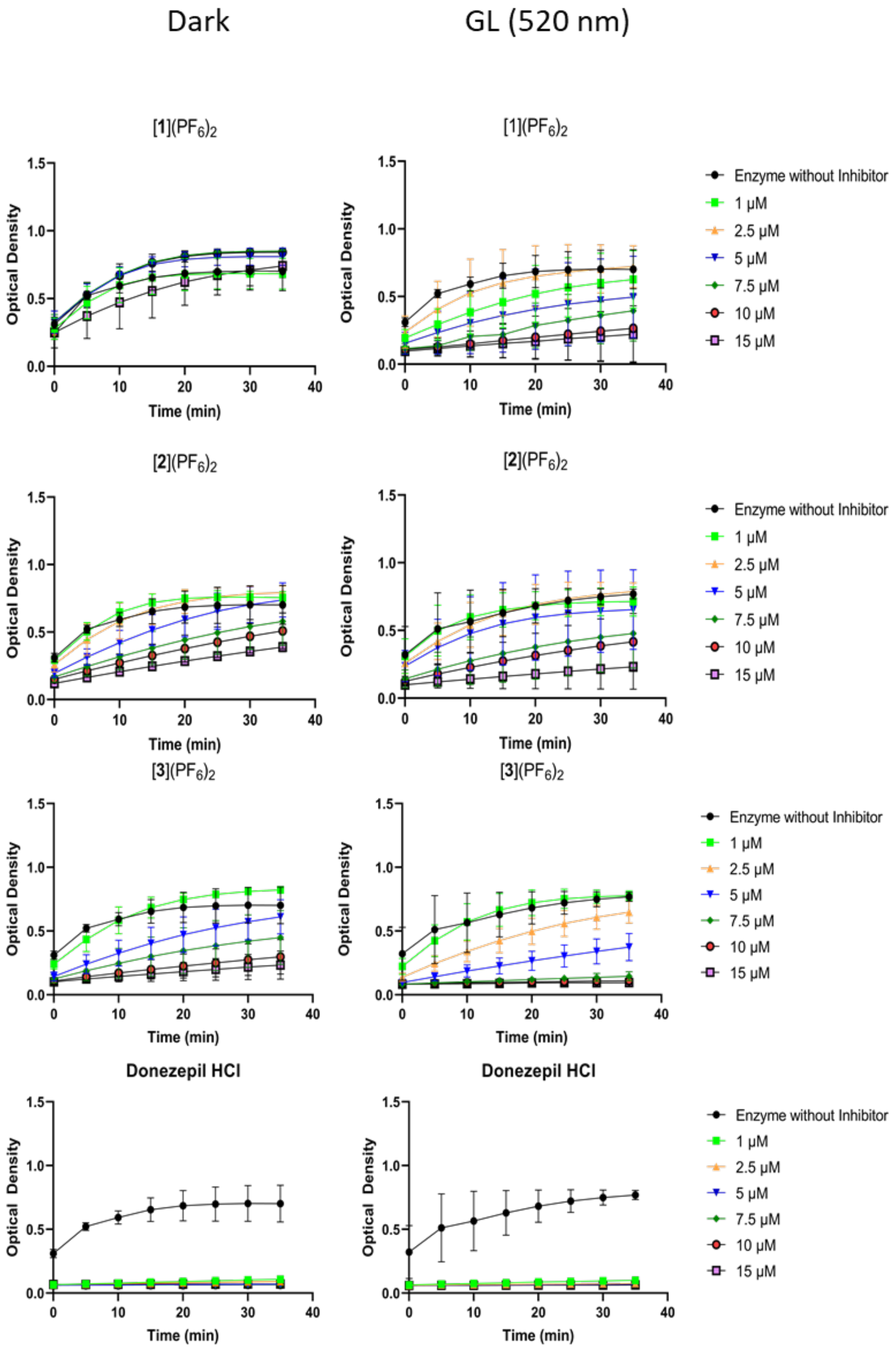

Figure S4. Optical density of DTNB conversion to TNB of [**1**]$(PF_6)_2$ – [**3**]$(PF_6)_2$ and Donezepil HCl at different concentrations (1, 2.5, 5, 7.5, 10 and 15 μM) incubated on the isolated AChe enzyme for 30 min in dark (left) or 30 min with green light (right) irradiation (520 nm, 25.2 J/cm^2). The reaction was initiated by the addition of DTNB and optical density was measured every 5 min for 35 min.

6. Computational Section

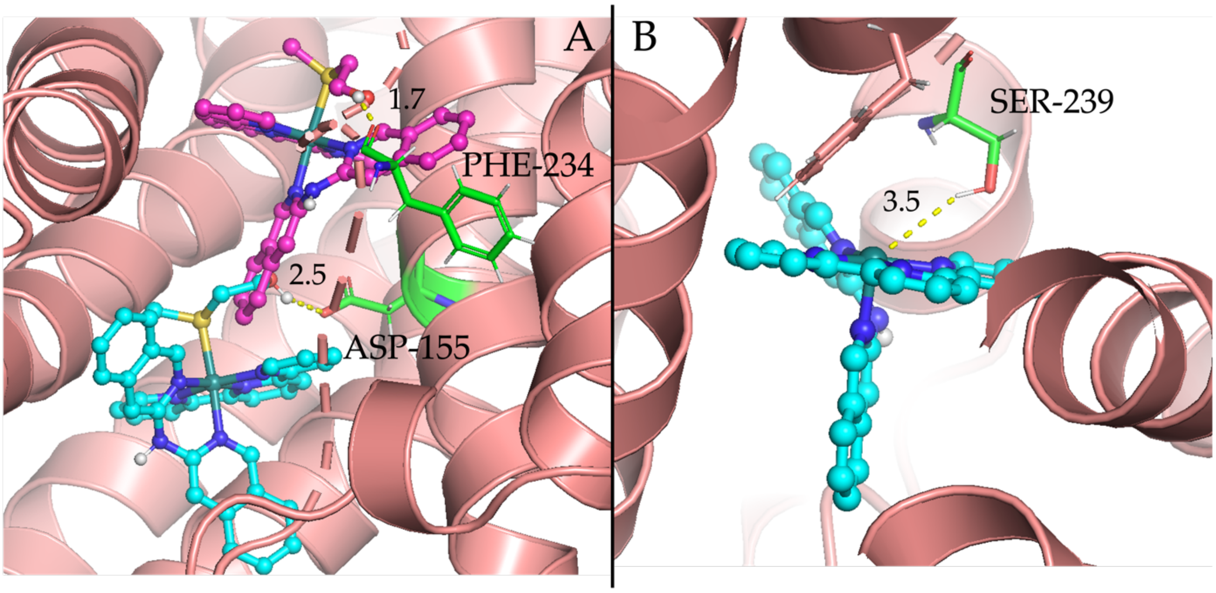

Figure S5. Poses obtained after docking compound [**3**]$^{2+}$ (A) and [**5**]$^{2+}$ (B) in the pocket of the serotonin receptor (PDB: 7WC4). From these docking simulations we observe that he hydroxyl group of the Hmte ligand of [**3**]$^{2+}$ forms a hydrogen bond with the carbonyl of the backbone of PHE-234 (purple) or the carboxylate of ASP-155 (cyan). Compound [**5**]$^{2+}$ exhibits long-range electrostatic and van der Waals interactions with the hydroxyl group of SER-239. In MetalDock, metal atoms are treated as hydrogen bond donors, and the observed H-bond interaction (in yellow) likely represent a strong attraction of Ru^{2+} to the oxygen atom. Since MetalDock does not explicitly account for electrons, it cannot simulate bond formation or cleavage. However, under physiological conditions a metal-assisted deprotonation of SER-239 may occur, after which it would become possible for ruthenium to form a coordination bond with SER-239. Distances are reported in Angstrom (Å). Residues that form an interaction with the compounds are highlighted in green.

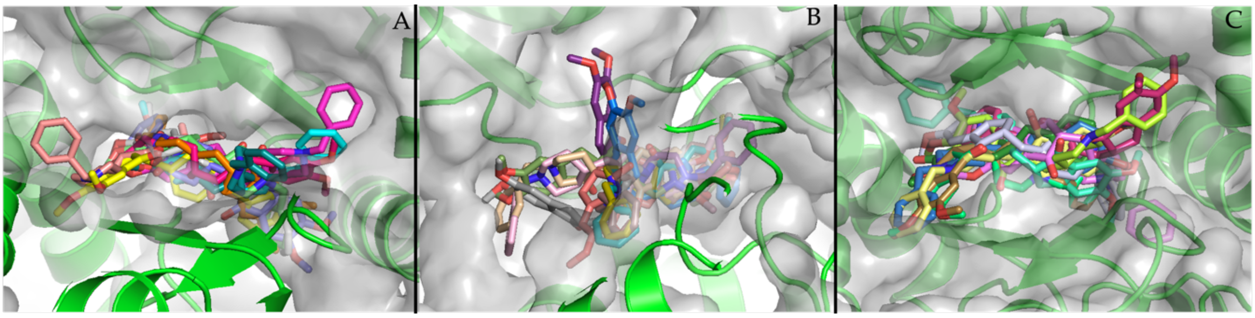

Figure S6. Images showing all ten docked poses of Donezipil HCl interacting with the NMDA receptor (A) (PDB:7EOR) and the AMPA receptor (B-C) (B: PDB:4LZ5, C: PDB:5YBG). Each pose predominantly exhibits van der Waals interactions, with the majority fitting nicely in the binding pocket.

7. References

(1) Busemann, A.; Flaspohler, I.; Zhou, X.-Q.; Schmidt, C.; Goetzfried, S. K.; van Rixel, V. H. S.; Ott, I.; Siegler, M. A.; Bonnet, S. Ruthenium-Based PACT Agents Based on Bisquinoline Chelates: Synthesis, Photochemistry, and Cytotoxicity. *JBIC J. Biol. Inorg. Chem.* **2021**, *26* (6), 667–674. https://doi.org/10.1007/s00775-021-01882-8.
